# Lignin Nanoparticles Containing Cobalt‐Cyanine Complexes: Potential Multifunctional Platforms for Photoacoustic Imaging and Photothermal Treatment of Bacterial Biofilms in Chronic Wounds

**DOI:** 10.1002/mabi.202500532

**Published:** 2026-02-03

**Authors:** Giulia Crivello, Matteo Felice Pezzuto, Paolo Armanetti, Claudio Cassino, Chiara Ceresa, Letizia Fracchia, Claudia Catarinicchia, Stefania Villani, Pietro Alifano, Christian Demitri, Luca Menichetti, Tzanko Tzanov, Gianluca Ciardelli, Clara Mattu

**Affiliations:** ^1^ Department of Mechanical and Aerospace Engineering Politecnico di Torino Torino Italy; ^2^ Institute of Clinical Physiology National Research Council Pisa Italy; ^3^ Department of Science and Technological Innovation Università del Piemonte Orientale “A. Avogadro” Alessandria Italy; ^4^ Department of Pharmaceutical Sciences Università del Piemonte Orientale “A. Avogadro” Novara Italy; ^5^ Department of Engineering for Innovation University of Salento Lecce Italy; ^6^ Department of Experimental Medicine University of Salento Lecce Italy; ^7^ Group of Molecular and Industrial Biotechnology Department of Chemical Engineering Universitat Politècnica De Catalunya Terrassa Spain

**Keywords:** antibiofilm, anti‐inflammatory, composite nanoparticles, photoacoustic imaging, photothermal therapy, phthalocyanine, wound regeneration

## Abstract

Chronic wounds (CWs) are characterized by persistent inflammation and bacterial biofilms, which hinder healing and contribute to antibiotic resistance. Therefore, innovative treatments with both anti‐inflammatory and antibiofilm properties are urgently needed. Here, cobalt phthalocyanine (CoPc), a photo‐excitable dye, is combined with polyphenolic lignin to develop CoPc‐Lig nanoparticles (NPs). These NPs demonstrate antioxidant activity by scavenging reactive oxygen species and inhibiting key enzymes implicated in CW pathophysiology. Moreover, they are internalized into *Staphylococcus aureus* and *Pseudomonas aeruginosa* biofilms, a critical feature for enhancing antibacterial effects. Upon near‐infrared light excitation, CoPc‐Lig NPs produce a thermal increase, which reduces bacterial viability and disrupts biofilm integrity. This mild photothermal effect is particularly advantageous in CW treatment, as excessive temperatures can damage newly formed tissue. Additionally, the NPs exhibit strong photoacoustic (PA) properties, enabling their use in PA imaging, an emerging non‐invasive technique for real‐time monitoring. The PA signal remains stable over time and is detected in ex vivo tissue phantoms. These findings highlight the potential of CoPc‐Lig NPs as a theragnostic platform for CW management, integrating antimicrobial cobalt, antioxidant polyphenols, and photo‐excitable phthalocyanines. Future studies will focus on optimizing photothermal treatment conditions and exploring synergies with debridement and antibacterial agents to enhance therapeutic outcomes.

## Graphical abstract

CoPc is combined with a lignin‐based material to obtain NPs sensitive to NIR irradiation and capable of ROS scavenging. Through the NPs, mild photothermal therapy can be achieved to act against bacterial biofilms.

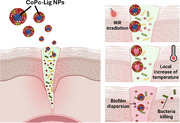

## 1 Introduction

Chronic wounds (CWs) are defined as wounds that fail to progress through a normal and timely healing process [1]. These wounds represent a significant health concern as they may lead to severe tissue damage and even death [2]. CWs are characterized by elevated levels of proinflammatory cytokines, reactive oxygen species (ROS), and enzymes (e.g., matrix metalloproteases, MMPs) that prevent wound closure by degrading the extracellular matrix (ECM) [3, 4]. Additionally, CWs are often colonized by multiple bacterial species organized into complex communities embedded in a self‐secreted matrix of extracellular polymeric substances (EPS), called biofilms [5]. Such bacterial communities are more difficult to eliminate than free bacteria because of their firm adhesion to tissues and the EPS matrix, which poses a physical barrier to the penetration of antimicrobials and host immune cells (e.g., neutrophils and macrophages) [6, 7, 8]. Studies indicate that biofilms in CWs are heterogeneous, with the coexistence of four to nearly twenty different bacterial species per wound [9, 10]. The prevalent species found in CWs biofilms are the Gram‐positive *Staphylococcus aureus* and the Gram‐negative *Pseudomonas aeruginosa* [8, 11, 12, 13], which are of particular concern, given the increasing number of antibiotic‐resistant strains [14, 15, 16, 17]. The peculiar phenotypic state of bacteria in biofilms, known as the sessile state, further exacerbates the development of antibiotic resistance. Indeed, the slow growth rate of sessile microorganisms restricts the action of antibiotics that target rapidly dividing cells, such as Penicillin [18]. The reduced antibiotic effect, coupled with the genetic diversity and cooperation of bacteria in heterogeneous biofilms, results in the development of antibiotic resistance [5, 7, 12, 19, 20]. The perpetuation of inflammation also increases capillary permeability and, subsequently, nutrient supply to bacteria, in turn favoring their proliferation and survival [7]. For example, the synergistic action of *S. aureus* and *P. aeruginosa* has been shown to produce persistent biofilms that delay wound healing and worsen the clinical outcomes [17, 21, 22]. Considering the deleterious effect of biofilms on wound healing and that their prevalence in CWs is estimated to be above 60% [11, 23], the development of new broad‐spectrum antibacterial treatments that actively induce biofilm dispersion and eradication is crucial [24].

The gold standard for infected wound treatment is surgical debridement coupled with antibiotic therapy, as after debridement, bacteria become more susceptible to antibacterial compounds [25]. However, biofilms are difficult to remove as they are tightly adherent to the wound bed. Moreover, debridement also removes newly formed tissue, thus negatively interfering with wound regeneration [24].

Therefore, therapeutic agents capable of acting specifically on biofilms without damaging healthy tissue are needed. To date, several therapeutic options targeting biofilms are available. [26] For instance, quorum‐sensing (QS) inhibitors, such as furanone C30 and RNA‐III‐inhibiting peptide, impair the chemical signaling within the biofilm, blocking its formation. However, the efficacy of this strategy on mature biofilms is limited [25]. Additionally, these compounds do not actively kill bacterial cells and require the combination with antimicrobial agents [27]. As an alternative, antimicrobial peptides, such as the human cathelicidin LL‐37, can be used to alter the stability of bacterial membranes, thus actively eradicating bacteria, preventing biofilm formation, or inducing its disruption [28]. However, peptides are expensive and prone to degradation in the CWs environment, rich in proteases. EPS degraders, such as dispersin B, deoxyribonuclease I (DNase I), and acylase, have also been proposed to target biofilms. These compounds alter the biofilm integrity by acting specifically on EPS components. Their action exposes bacteria by lowering EPS protection, making them more susceptible to antibacterial agents [29]. In recent years, treatments based on physical removal, without debridement, have also been explored for the elimination of biofilms. These methods exploit ultrasound, electric field, light irradiation, or magnetic field to stimulate biofilm detachment or disaggregation [30, 31]. For instance, Dong et al. [32], proposed magnetic NPs that induced biofilm disruption through mechanical friction under an external magnetic field. These NPs could be remotely activated, resulting in biofilm removal within narrow tubes, such as catheters. Photothermal therapy (PTT) is another promising treatment against biofilms. In PTT, a photo‐excitable molecule is stimulated with near‐infrared (NIR) light, resulting in increased local temperature and thermal ablation of target cells [33, 34]. This strategy has been used to remotely control biofilm eradication on titanium implants in vivo [35]. However, heat generation may damage healthy tissue [35]. Therefore, a combination of mild PTT with the local release of antimicrobial compounds has been proposed to enhance treatment efficacy while reducing thermal damage [36, 37]. For instance, Zhao et al. [36], proposed thermosensitive liposomes encapsulating the antibiotic drug tobramycin. Upon NIR irradiation, a photosensitive cyanine dye co‐encapsulated with tobramycin produced a local increase in temperature to 45°C, which stimulated tobramycin release and potentiated its anti‐biofilm activity in vitro and in vivo.

Based on the above considerations, the development of multifunctional treatments able to reduce the inflammatory state and the microbial and biofilm burden is highly needed. In recent years, different nanomaterials have been developed showing promising antioxidant and antibacterial properties [4, 26]. Among these, natural polyphenols have raised interest for their ROS scavenging properties and biocompatibility, while metal ions have been largely studied for their intrinsic antibacterial activity [4, 26]. In our previous publication [38], we designed lignin‐based nanoparticles (NPs) capable of mitigating the inflammatory states of CWs by virtue of the intrinsic anti‐inflammatory capacity of the material. When loaded with cobalt, these NPs exerted antimicrobial action on bacteria but did not act on preformed biofilms. Herein, to further expand the activity and applicability of lignin‐based NPs, we propose lignin NPs entrapping cobalt‐phthalocyanine (CoPc) complexes for the photothermal ablation of biofilms and photoacoustic (PA) imaging in CWs. Phthalocyanines (Pc) are photosensitive compounds presenting absorbance in the NIR spectrum, used in clinical trials as photosensitizers for photodynamic treatment (PDT) or photoimmunotherapy [39]. In this work, a commercially available CoPc complex was combined with a lignin (Lig)‐tannic acid (TA)‐based material to obtain CoPc‐Lig NPs with anti‐inflammatory and photothermal properties. Additionally, under nano‐second pulsed laser NIR excitation, Pc can produce PA signals, which can be exploited for non‐invasive monitoring of the healing process in the constantly evolving CW environment. In recent years, dressings capable of giving real‐time feedback on wound development [69] based on temperature, pH, and glucose levels variations have been proposed [40, 41, 42, 43, 44]. These dressings either change colour or incorporate sensors that can monitor specific parameters. However, these solutions are still in an early stage of development. PA imaging is a non‐invasive imaging modality with the potential to identify and detect inflammation [45, 46, 47]. To the best of our knowledge, PA imaging has not been used in the monitoring of CWs. However, given its advantages, PA imaging is a promising approach to evaluate wound progression in real time.

## 2 Results and Discussion

### 2.1 Synthesis and Characterization of Lig‐TA

**FIGURE 1:**
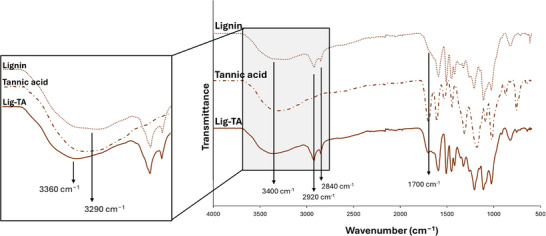
FT‐IR spectrum of Lig‐TA compared to the spectra of Lignin and tannic acid. Peaks at 2920 cm^−1^ and 2840 cm^−1^ were attributed to the C─H stretching of methyl groups of lignin. The peaks at 1700 cm^−1^ were attributed to the C═O stretching vibration of unconjugated carbonyl groups present both on lignin and tannic acid. The band around 3400 cm^−1^ was assigned to the stretching of the OH‐bond on the aromatic ring.

A Lig‐based material enriched in polyphenols was successfully synthesized through the enzymatic conjugation of TA to Lig, resulting in an enhanced antioxidant effect. Indeed, the phenolated lignin (Lig‐TA) showed an increase in GAE per mg of conjugate compared to pristine lignin (150 ± 7 µg GAE/mg for Lig‐TA vs. 120 ± 2 µg GAE/mg for lignin, p = 0.013). Successful TA conjugation was also confirmed by ATR‐FTIR. The ATR‐FTIR spectrum of Lig‐TA presented two peaks at 2840 cm^−1^ and 2920 cm^−1^, related to the C─H stretching of methyl groups characteristic of Lig, confirming the presence of Lig in the newly obtained material. Moreover, the Lig‐TA spectrum presented a clear peak at 1700 cm^−1^ (Figure 1) attributed to the C═O stretching vibration of unconjugated carbonyl groups. These groups are present in both Lig and TA, but are more abundant in TA, resulting in a more intense peak. Therefore, the more evident peak in Lig‐TA suggests the presence of TA in the conjugated material. Additionally, a clear shift in the band at 3400 cm^−1^ (from 3290 cm^−1^ to 3360 cm^−1^), attributed to the stretching of the OH‐bond on the aromatic ring [48, 49], suggests that the cross‐linking reaction between Lig and TA occurred during laccase‐assisted phenylation [50].

### 2.2 Synthesis and Characterization of CoPc‐Lig NPs

**FIGURE 2:**
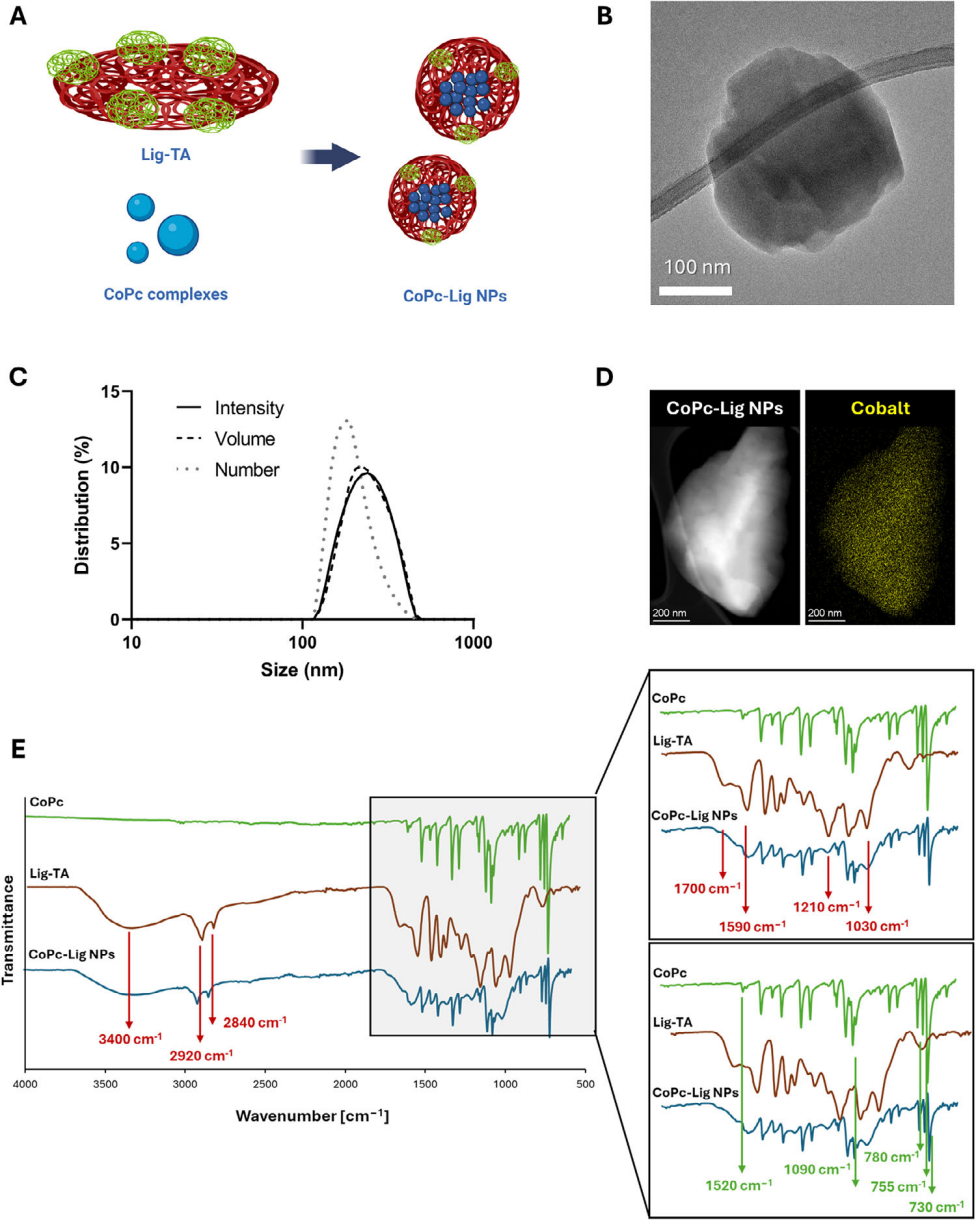
CoPc‐Lig NPs preparation and characterization. A) Schematic of the CoPc‐Lig NPs structure. B) TEM micrograph of CoPc‐Lig NPs showing an irregular round morphology. C) Intensity (solid line), volume (dashed line), and number (dot line) size distributions of CoPc‐Lig NPs. D) TEM micrographs coupled with EDX elemental mapping showing the distribution of Cobalt (yellow) in the NPs matrix. E) ATR‐FTIR spectra of the CoPc‐Lig NPs (Blue) compared to the spectrum of bare Lig‐TA (red) and CoPc (green). Red arrows indicate Lig‐TA‐related peaks, while green arrows indicate CoPc‐related peaks.

CoPc‐Lig NPs were synthesized by combining CoPc and Lig‐TA as schematized in Figure 2A. Lig was expected to interact with Pc through hydrophobic and π−π stacking interactions, facilitated by the aromatic groups on both compounds, thus forming nanoparticles [49, 51]. CoPc‐Lig NPs with an irregular round shape (Figure 2B) were successfully obtained at a LigTA/CoPc mass ratio of 3:2, with a production yield of 6.5%. This LigTA/CoPc ratio allowed a small diameter (around 250 nm) and a low PDI (0.14) (Table 1; Figure S1). Moreover, the number, intensity, and volume‐weighted size distributions showed overlapping profiles (Figure 2C), which confirmed a monodisperse distribution. The NPs presented a negative ζ potential of ‐32 mV, intermediate between the ζ potential values of CoPc complexes (−28 ± 0.3 mV) and that of Lig‐TA (−40 ± 0.8 mV), further confirming the interactions between the two components (Table 1). TEM micrographs (Figure 2D) coupled with EDX analysis with elemental mapping showed homogeneous distribution of cobalt within the NP structure (Figure 2C), while ICP‐MS analysis indicated a cobalt content of 15.7 µg/mg NPs (Table 1).

**TABLE 1:** Physicochemical properties of CoPc‐Lig NPs.

Size (nm)	PDI	ζ Potential (mV)	Co content (µg/mg NPs)	Yield (%)
247 ± 1	0.14 ± 0.03	−32 ± 0.5	15.7 ± 3.9	6.5

The ATR‐FTIR spectrum (Figure 2E) confirmed the presence of both Lig‐TA and CoPc in the NPs, as the characteristic peaks of Lig‐TA (Table 2) and CoPc (Table 3) were identified [52, 53, 54]. Interestingly, the peak at 3400 cm^−1^, corresponding to the stretching of the O‐H bond on the aromatic ring of Lig‐TA, appeared slightly shifted in CoPc‐Lig NPs, suggesting an interaction between the CoPc complex and the phenolic groups of Lig‐TA. The UV–vis spectrum of CoPc‐Lig NPs (Figure S1) further confirmed the presence of CoPc complexes in the NPs, as indicated by the two adsorption peaks at 630 nm and 710 nm, which were also found in the spectrum of bare CoPc complexes.

**TABLE 2:** ATR‐FTIR signals related to Lig‐TA.

Wavenumber (cm^−1^)	Lig‐TA related bonds
1030	OH stretching of primary alcohol
1210	Aromatic C─O stretching
1590	Aromatic ring vibration
1700	C═O stretching non‐conjugated carbonyl
2840–2920	C─H stretching of methyl groups

**TABLE 3:** ATR‐FTIR signals related to CoPc.

Wavenumber (cm^−1^)	CoPc‐related bonds
730	C─H out‐of‐plane deformation
755	Co─N bond vibration
780	C═N in plane stretching vibration
1090	C─H in‐plane deformation
1520	C═N stretching

### 2.3 Biological Characterization of CoPc‐Lig NPs

#### 2.3.1 Cell Internalization and Cytocompatibility of CoPc‐Lig NPs

**FIGURE 3:**
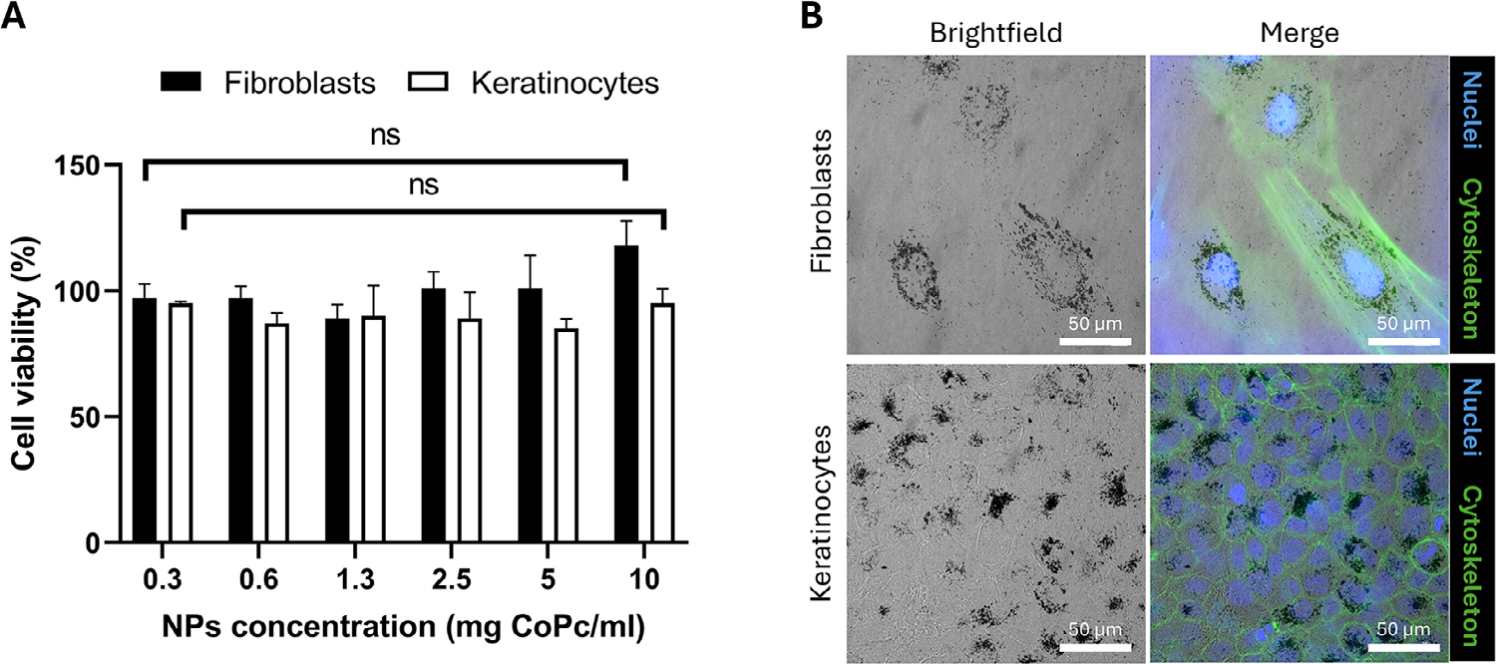
Cell compatibility of CoPc‐Lig NPs. A) Cell viability of fibroblasts (black bars) and keratinocytes (white bars) exposed to different concentrations of CoPc‐Lig NPs for 24 h. B) Confocal images showing cell internalization of CoPc‐Lig NPs (0.5 mg/mL) by fibroblasts and keratinocytes. The dark areas in brightfield images represent CoPc‐Lig NPs, while the fluorescence channels show cell nuclei (Blue), and cell cytoskeleton (green).

CoPc‐Lig NPs were tested on two CW‐relevant cell types, fibroblasts and keratinocytes. Cell viability assay showed no significant reduction of viable cells in comparison to untreated cells at any of the tested concentrations for both cell types, indicating excellent cytocompatibility (Figure 3A). The administered content of Co at the maximal NPs concentration of 4 mg/mL corresponded to 60 µg Co/mL. Previous studies reported the toxicity of cobalt ions and cobalt oxide NPs at concentrations between 5 µg/mL and 25 µg/mL [55, 56]. Our results suggest that the intrinsic toxicity of cobalt was markedly reduced in CoPc‐Lig NPs, probably due to cobalt coordination in the CoPc complex and to the presence of the Lig‐TA matrix. Additionally, confocal microscopy observations showed that CoPc‐Lig NPs were internalized by both cell types (Figure 3B) and appeared to be located around the cell nuclei.

### 2.4 Anti‐Inflammatory and Antibacterial Activity of CoPc‐Lig NPs

**FIGURE 4:**
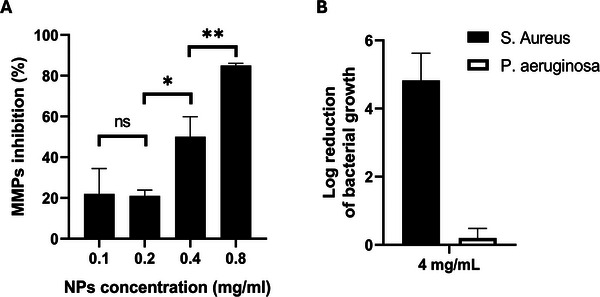
Anti‐inflammatory and antibacterial activity of CoPc‐Lig NPs. A) MMPs inhibition by CoPc‐Lig NPs at different concentrations. B) Antibacterial activity of CoPc‐Lig NPs obtained through CFU count for *S. aureus* (black bars) and *P. aeruginosa* (white bars) treated with CoPc‐Lig NPs at 4 mg/mL for 24 h.

CoPc‐Lig NPs exhibited high MMP inhibition capacity, even at low concentrations. Indeed, 50% MMP inhibition was achieved at 0.4 mg/mL, while over 80% inhibition was obtained at 0.8 mg/mL (Figure 4A). Moreover, the DPPH assay showed ROS scavenging activity already at a concentration of 0.2 mg/mL. The anti‐inflammatory properties observed in the NPs were mainly attributed to the presence of phenolic groups in LigTA, which are known for their high antioxidant and anti‐inflammatory potential [38], as no evidence in the literature suggests a direct anti‐inflammatory action of CoPc.

CoPc‐Lig NPs also displayed bacterial inhibition capacity against *S. aureus*. The CFU count showed a 4.8 log reduction for *S. aureus* at a CoPc‐Lig NPs concentration of 4 mg/mL (Figure 4B). On the other hand, no inhibition was observed for *P. aeruginosa* at the same NPs concentration. The difference in susceptibility between the Gram‐positive *S. aureus* and the Gram‐negative *P. aeruginosa* was attributed to their different cell membrane composition. Indeed, gram‐negative species present a double‐layered membrane, which makes their eradication more challenging [57]. Additionally, *P. aeruginosa* is known to possess lower membrane permeability compared to other Gram‐negative species, which further prevents the action of several antibiotics [58].

#### 2.4.1 Internalization of CoPc‐Lig NPs in Biofilms

**FIGURE 5:**
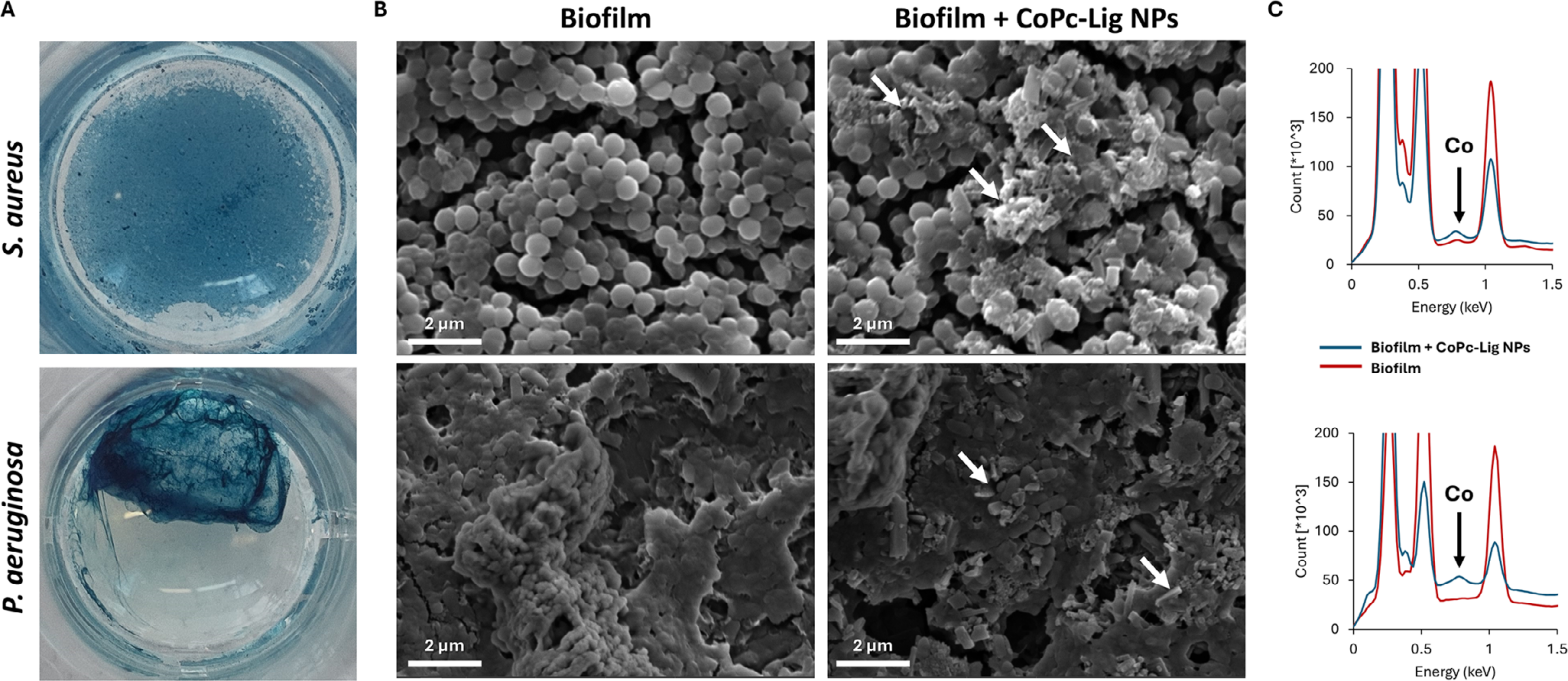
Internalization of CoPc‐Lig NPs into S. aureus and P. aeruginosa biofilms. A) Images of fixed biofilms visibly show the internalization of CoPc‐Lig NPs (blue coloration). B) SEM images of CoPc‐Lig NPs at 0.5 mg/mL. White arrows indicate agglomerates of NPs. C) EDX spectra showing the appearance of the Co peak in biofilms treated with CoPc‐Lig NPs (blue line) compared to untreated biofilms (red line) for *S. aureus* and *P. aeruginosa*.

As expected, CoPc‐Lig NPs did not exhibit antibiofilm activity *per se* (data not shown), as the antibiofilm capacity of these NPs should stem from their photothermal effect. CoPc‐Lig NPs were well internalized into both *S. aureus* and *P. aeruginosa* biofilms, as shown by the blue coloration associated with CoPc, assumed by both biofilms after treatment (Figure 5A). SEM micrographs confirmed the presence of CoPc‐Lig NPs in *P. aeruginosa* and *S. aureus* biofilms. Treated biofilms presented large agglomerates that were absent in the untreated counterparts (Figure 5B). These agglomerates were considered CoPc Lig NPs, as the EDX analysis indicated the presence of cobalt (Figure 5C). UV–vis spectroscopy confirmed successful internalization, particularly in *S. aureus* biofilms, for which nearly 30% internalization was achieved. CoPc‐Lig NPs possess a negative surface charge, which is not considered favorable for biofilm internalization. This high biofilm internalization could therefore be attributed to the adhesive properties of Lig, suggesting further investigation of the specific interactions between Lig and the EPs component of the biofilm [59, 60].

### 2.5 Photoacoustic and Photothermal Characterization of CoPc‐Lig NPs

#### 2.5.1 Photoacoustic Characterization

**FIGURE 6:**
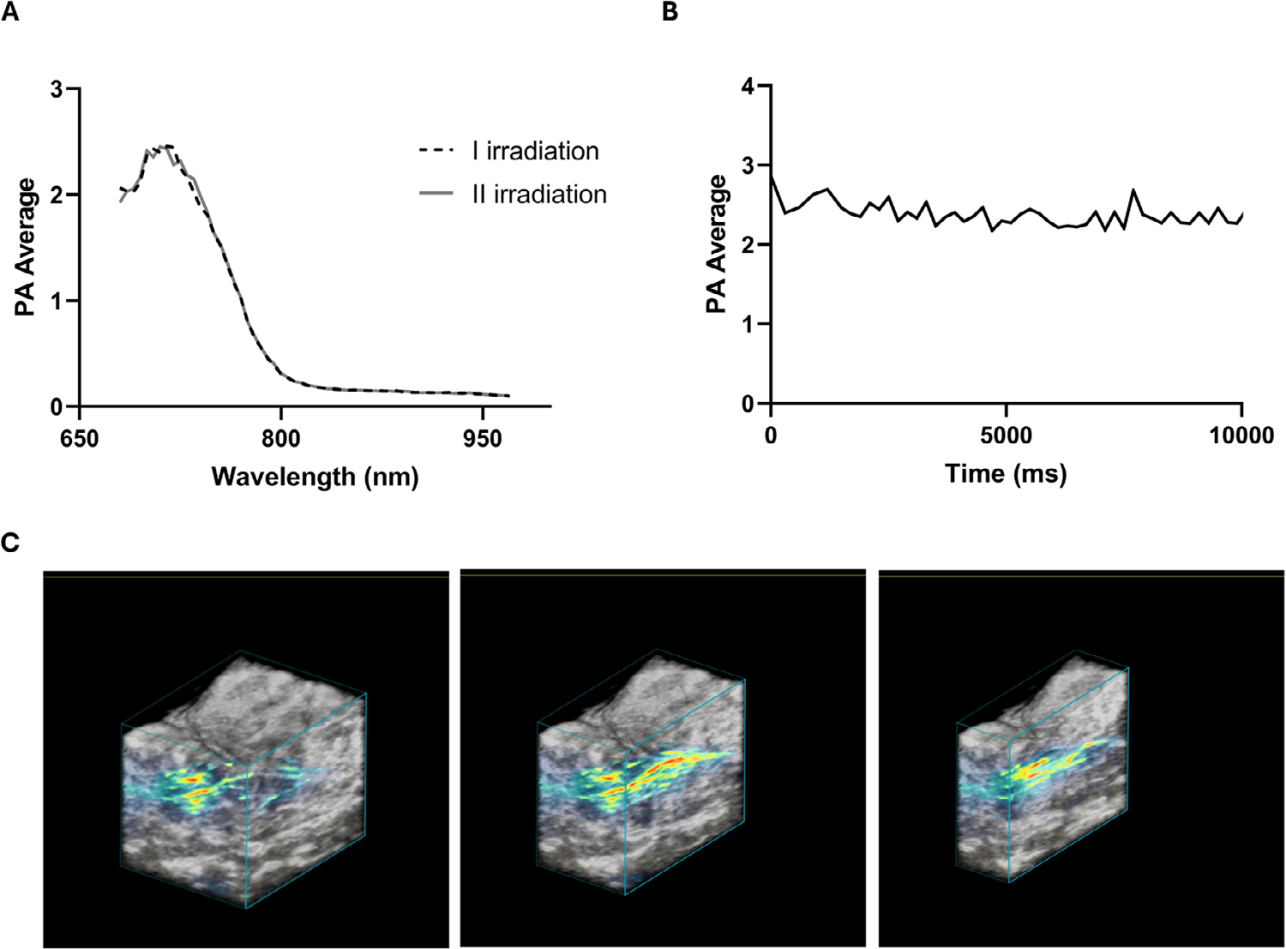
PA characterization of the CoPc‐Lig NPs in ex vivo phantoms. A) PA spectra of excitation at different wavelengths in two different moments: initially (I irradiation) and after prolonged pulsed laser illumination (II irradiation). B) PA stability under prolonged pulsed laser illumination at 710 nm. C) Representative sections of the 3D volume reconstruction showing the distribution of CoPc‐Lig NPs in the tissue (gray scale for US, rainbow scale for PA signal).

The PA activity of CoPc‐Lig NPs in aqueous suspension and in bovine ex vivo phantoms is reported in Figure S4 and Figure 6, respectively. Under both conditions, CoPc‐Lig NPs presented an evident PA effect, particularly for excitation wavelengths in the range of 700–715 nm (Figure 6A; Figure S4), consistent with their absorption profile. After injection in the ex vivo tissue phantom, no signal bleaching was observed under prolonged pulsed laser illumination at 710 nm (Figure 6B), confirming the robustness of the PA signal. These results demonstrate that CoPc‐Lig NPs maintain their photoacoustic properties under biologically relevant conditions, supporting their potential for PA imaging in complex media such as tissue‐mimicking environments. The 3D reconstruction of the ex vivo phantom (Figure 6C) highlights the distribution of CoPc‐Lig NPs within the tissue, showing that the particles permeated the surrounding area and were evenly distributed around the injection site. Quality parameters, such as Signal to Noise Ratio (SNR) and Contrast to Noise Ratio (CNR) were measured to be 36 and 33 dB, in line with other contrast agents used in photoacoustic imaging [61, 62]. The PA signal stability, assessed by calculating the percentage coefficient variation (%CV), showed PA signal fluctuations of 2.7%, indicating stable imaging performances over time [63]. Such high stability over time suggests that the proposed platform can be used for multiple stimulations, supporting repeated treatment regimens.

#### 2.5.2 Photothermal Activity Within Biofilm

**FIGURE 7:**
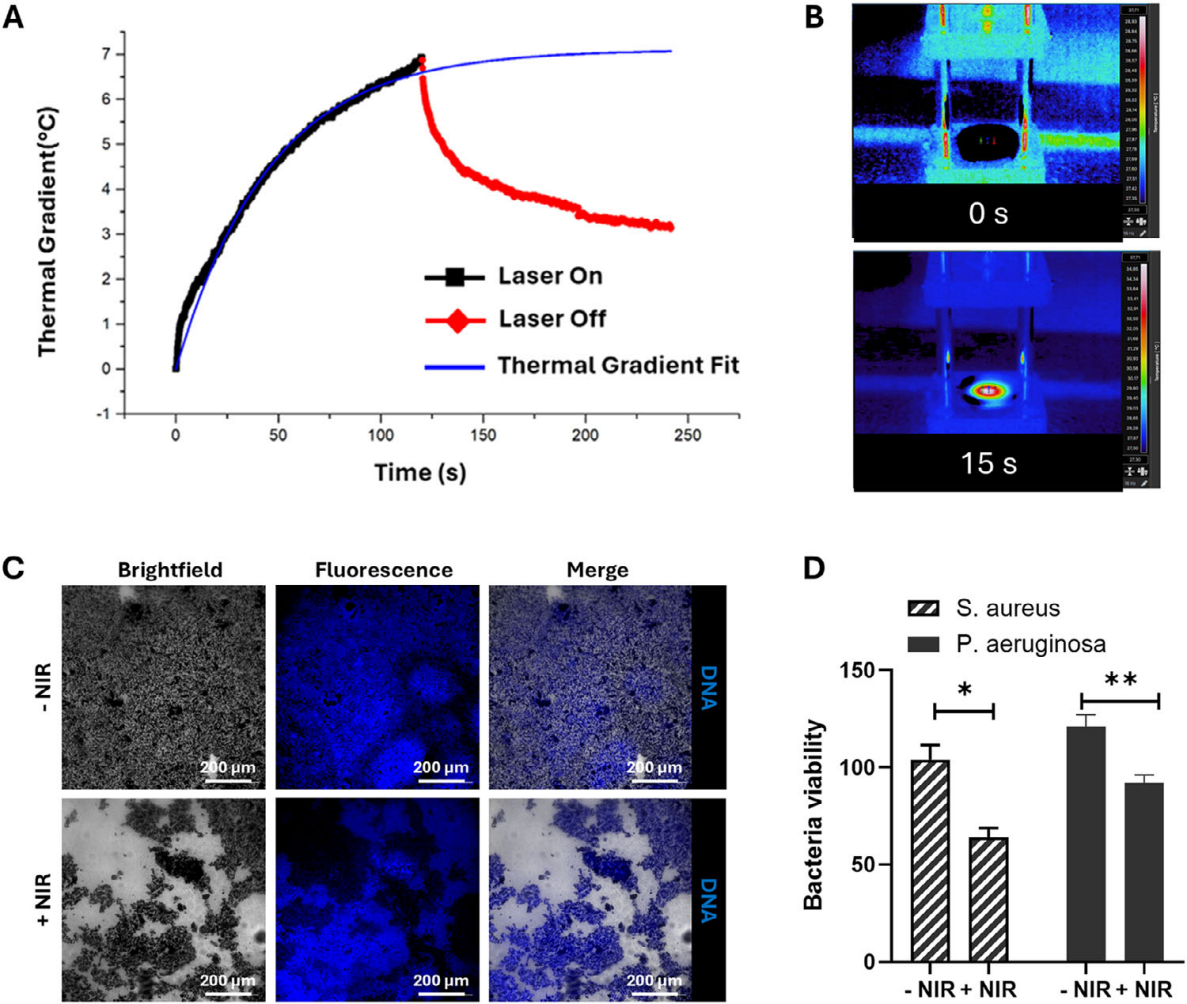
Photothermal activity of CoPc‐Lig NPs within treated biofilm. A) The graph presents the increase in temperature following NIR light excitation (black line) of the CoPc‐Lig NPs internalized into *S. aureus* biofilms. After two minutes of irradiation, a temperature increase of 7°C was observed. The temperature rapidly decreased when the NIR stimulation was removed (red line). Using these data, it was possible to obtain a thermal gradient fit indicating the expected increase in temperature if the laser is maintained active for a longer time (Blue line). B) Images of the irradiated biofilm treated with NPs obtained through a thermal camera. C) Confocal images showing the morphology of the *S. aureus* biofilm before (− NIR) and after (+ NIR) treatment with NIR irradiation. Brightfield images show CoPc‐Lig NPs, fluorescence images show the bacterial DNA, and the merged images show the colocalization of NPs and bacterial cells. D) S. aureus and P. aeruginosa viability within biofilm treated with CoPc‐Lig NPs before (− NIR) and after (+ NIR) treatment with NIR irradiation. The viability was assessed immediately after treatment.

To study the photothermal effect of CoPc‐Lig NPs, NPs were first internalized inside the biofilm, followed by NIR irradiation and temperature monitoring. The *S. aureus* biofilm treated with CoPc‐Lig NPs showed a 7°C increase in temperature after 2 min of irradiation at 660 nm (Figure 7A,B), suitable for mild heat treatment [36, 64], while the untreated biofilm did not register significant temperature variations under the same irradiation conditions (Figure S5). Immediately after NIR irradiation, bacterial viability decreased by 40% and 30% for *S. aureus* and *P. aeruginosa*, respectively (Figure 7D). As a result, the integrity of the *S. aureus* biofilm was altered Figure 7C after NIR irradiation, as evidenced by the presence of several gaps with no cells. Therefore, treatment with NIR actively reduced biofilm integrity and promoted its disruption. This increased fragility was also perceived during sample preparation and could facilitate the mechanical removal of the biofilm through debridement. This result is promising since it proves that photothermal treatment with the CoPc‐Lig NPs affected the bacteria within the biofilm. Nevertheless, NIR treatment was not sufficient to kill bacteria and to eliminate the biofilm. Indeed, 24 h after treatment, the viability of both biofilms was restored (Figure S6).

### 2.6 Photothermal Activity of CoPc‐Lig NPs in *G. mellonella*

**FIGURE 8:**
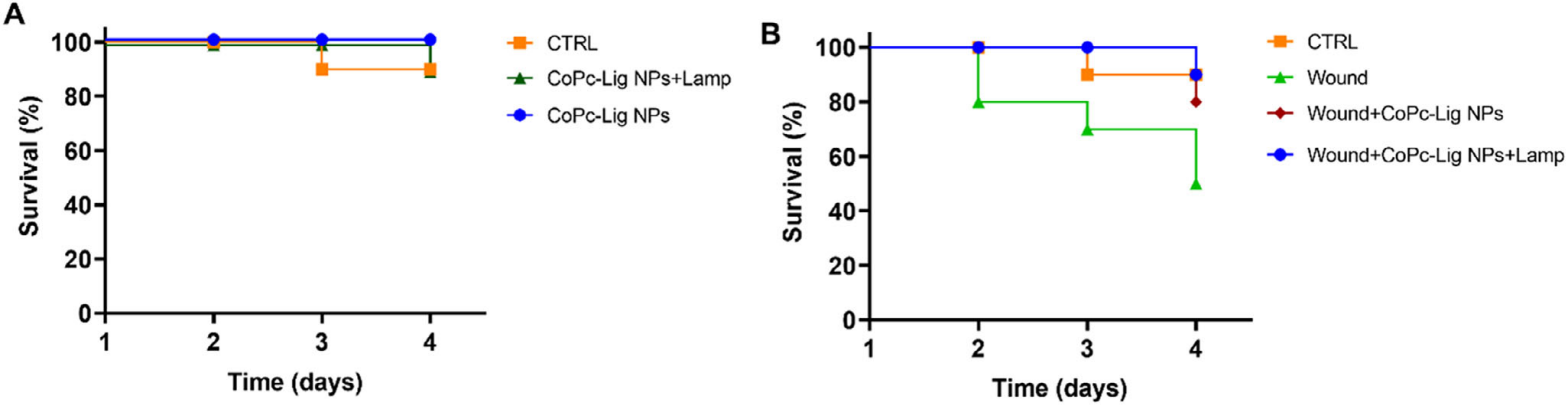
Kaplan‐Meier survival curves of *G. mellonella* larvae treated with CoPc‐Lig NPs. Survival rate of A) larvae with intact skin and B) larvae with a wound, treated with CoPc‐Lig NPs with and without irradiation.

The biocompatibility and safety of the CoPc‐Lig NPs used for photothermal therapy were assessed in a simplified wound model in the G. mellonella larvae. This model represents an alternative to conventional mammalian models, providing a reliable tool for validating in vitro findings [65, 66]. The Kaplan‐Meier survival curves reported in Figure 8 clearly indicate that the administration of CoPc‐Lig NPs, followed by irradiation with a 660 nm lamp, was well tolerated in larvae with intact skin (Figure 8A). In the case of injured skin (Figure 8B), the application of CoPc‐Lig NPs produced a significant improvement in survival, with 80% survival in the treated group compared to 50% in the untreated controls (Wound). Furthermore, when CoPc‐Lig NPs were irradiated, the survival was 90% at day 4, indicating that photoactivation did not compromise the safety of the NPs, leading to a moderate improvement in the survival rate in comparison to bare CoPc‐Lig NPs. The improvement in survival following excitation suggested that the treatment mechanism may involve photodynamic activation, which may promote cell proliferation and prevent bacterial infections [67].

#### 2.6.1 Photoacoustic Activity Within *S. aureus* Biofilm

**FIGURE 9:**
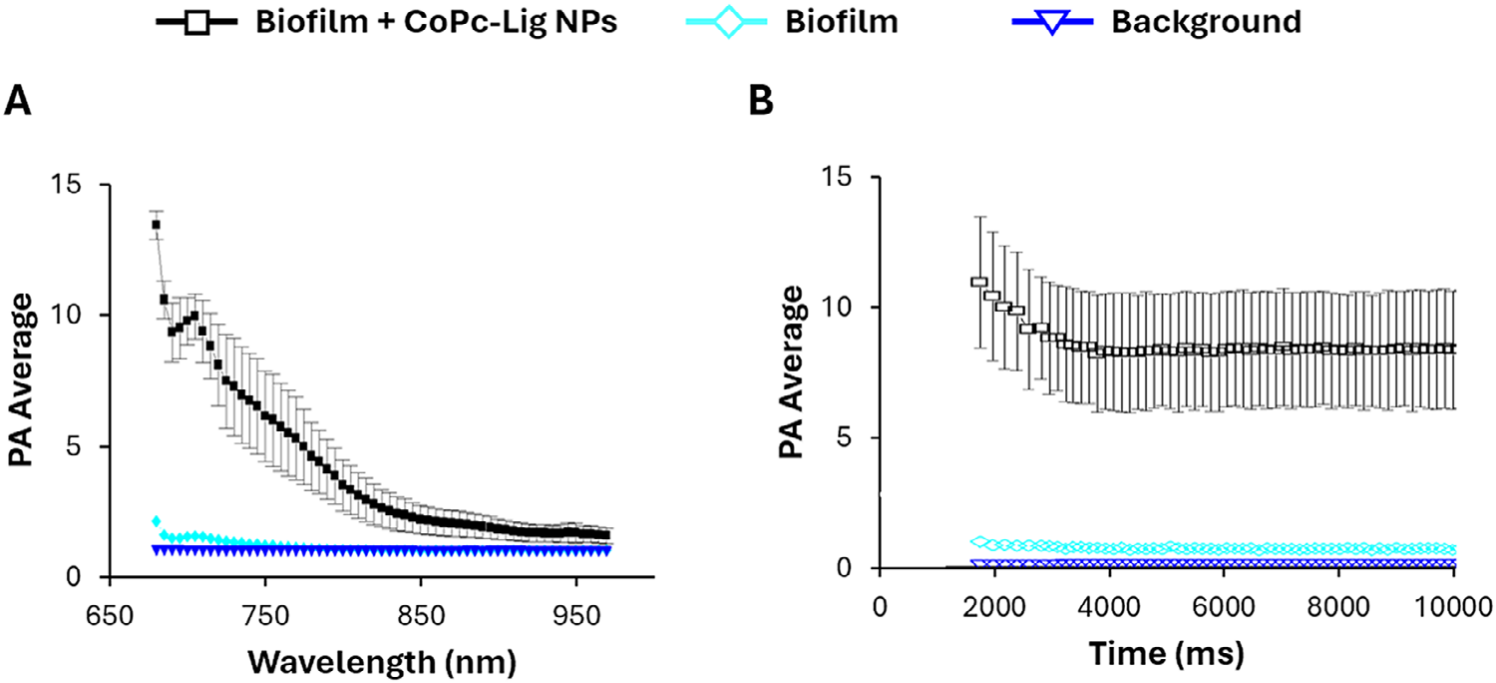
PA activity of CoPc‐Lig NPs internalized into *S. aureus* biofilm. A) PA spectra of excitation of CoPc‐Lig NPs at different wavelengths. B) PA stability of CoPc‐Lig NPs under continuous irradiation at 710 nm.

The PA activity of the NPs was assessed within *S. aureus* biofilm. The bacterial biofilms were grown on 0.5 cm‐thick custom‐made polydimethylsiloxane slides. After incubation with CoPc‐Lig NPs, an additional PDMS layer was poured on the biofilms to facilitate the PA analysis. Similarly to observations in ex vivo phantoms, CoPc‐Lig NPs in the biofilm presented an evident PA effect, which remained stable over time, with a maximum signal for excitation wavelengths in the range of 700–715 nm (Figure 9). These properties are promising for expanding the applicability of CoPc‐Lig NPs to PA imaging in CWs.

## 3 Discussion

CWs are complex pathological conditions marked by a failure in the normal healing process [1]. They are characterized by persistent inflammation, elevated levels of ROS, and overexpression of MMPs, which degrade the ECM and prevent tissue repair [3]. Therefore, systems capable of scavenging ROS and inhibiting MMP activity may help mitigate the chronic inflammatory state. Additionally, CWs are frequently colonized by multiple bacterial species that form biofilms, which increase resistance to treatment and hinder healing [5]. Herein, we combined polyphenolic Lig‐TA with CoPc to obtain CoPc‐Lig NPs with the ability to reduce inflammation, minimize ECM degradation by inhibiting ECM‐degrading enzymes, and reduce biofilm integrity upon NIR irradiation.

The obtained NPs showed small size, spherical morphology, and excellent cytocompatibility even at high concentrations (Table 1; Table S1). Moreover, NPs presented the desired structure, combining polyphenolic Lig‐TA with CoPc, as demonstrated by FTIR‐ATR, UV–vis and TEM/EDX analyses (Figure 2 and Tables 2 and 3; Figures S1–S3). The NPs' production yield was 6.5%, which is not ideal for scale‐up production of these NPs. Our results showed that the lignin‐based material is the main component lost in the process, and the UV–vis analysis in Figure S7 clearly showed that only Lig‐TA is eliminated during the first centrifugation step. Future tests should focus on improving the efficiency of the process toward its scalability. For instance, reducing the Lig‐TA:CoPc ratio may help improve the process yield, while reusing the unreacted lig‐TA recovered from the first centrifugation step could enhance the process efficiency. Polyphenols are known to exert antioxidant and anti‐inflammatory effects, due to the radical scavenging and zinc chelation ability of their hydroxyl groups [68, 69, 70, 71]. Due to their therapeutic potential, plant‐derived phenolic compounds have been incorporated into wound dressings with promising results, supporting tissue regeneration and accelerating the healing process [68, 72, 73, 74]. For instance, Díaz‐González et al. [68]. confirmed that a polyphenolic plant extract from *Hamamelis virginiana* possessed strong antioxidant and MMPs inhibition activity, as well as a chelating effect of zinc ions, needed for MMP activity. In our previous work, we also showed that the polyphenolic nature of Lig‐TA reduced the oxidative stress and inhibited enzymes overexpressed in CWs [38]. Notably, we showed that polyphenolic NPs displayed enhanced enzyme‐inhibitory effect in comparison to the bulk material, due to their higher surface area, which enhanced the interaction with target enzymes [38]. In this study, CoPc‐Lig NPs maintained potent antioxidant activity and MMP inhibition capacity, which were attributed to the organic polyphenolic core of the particle (Figure 4).

CoPc was encapsulated for its dual function, combining photothermal conversion, enabled by the phthalocyanine cage, with the antimicrobial properties given by the cobalt core [38]. Indeed, we observed a 4.8 log reduction in the CFU count for *S. aureus* after treatment with CoPc‐Lig NPs at a concentration of 4 mg/mL, which corresponds to an equivalent cobalt concentration of 60 µg/mL (Figure 4). On the other hand, no inhibition was observed for the Gram‐negative *P. aeruginosa*. We previously showed that cobalt‐loaded NPs were able to inhibit bacterial activity for both *S. aureus* and *P. aeruginosa* at equivalent cobalt concentrations of 50 µg/mL and 100 µg/mL, respectively [38]. Thus, the results for CoPc‐Lig NPs align with our prior observations for *S. aureus*, suggesting that higher concentrations may also produce an inhibitory effect on *P. aeruginosa*. Interestingly, cobalt‐loaded NPs presented cytotoxicity in human fibroblasts and keratinocytes above an equivalent cobalt concentration of 25 µg/mL. Herein, CoPc‐Lig NPs showed cytocompatibility even at 60 µg/mL of equivalent cobalt concentration (Figure 3). This suggests that the cytotoxic activity of cobalt was reduced in CoPc‐Lig NPs, probably due to its coordination in the CoPc complex and to the presence of the Lig‐TA matrix. The preliminary in vivo tests in the simplified G. mellonella larva confirmed the safety of the CoPc‐Lig NPs, further supporting their applicability (Figure 8).

Despite their negative surface charge, CoPc‐Lig NPs were easily internalized into bacterial biofilms with a 30% efficiency, suggesting interactions between CoPc‐Lig NPs and the EPS in biofilm. Future testing should focus on better investigating the nature of these interactions and the components involved, to clarify the role of the NPs as anti‐biofilm agents and to support the design of new and more efficient platforms for biofilm eradication (Figure 5). As expected, CoPc‐Lig NPs did not display intrinsic antibiofilm activity, since CoPc lacks this function without NIR activation [36]. On the other hand, upon NIR excitation, the NPs generated a controlled thermal increase of 7°C, with local temperature not exceeding 50°C (Figure 7; Figure S5). This mild heat reduced bacterial viability up to 40% and disrupted the biofilm integrity, while NIR irradiation without NPs did not influence bacterial viability within biofilms. This mild photothermal therapy (PTT) is particularly advantageous in CWs, where normal cells and bacteria are heavily intertwined, as high‐temperature PTT (>50°C) may damage the host tissue through protein denaturation and induce cytotoxicity, in turn impeding healing [75]. In contrast, mild hyperthermia (42–45°C) has been shown to enhance the efficacy of antimicrobial agents [36, 64] and to promote tissue regeneration by stimulating endothelial cell maturation and vascularization [76, 77]. Several studies combining mild PTT with drug delivery demonstrated successful biofilm treatment [36, 37]. For instance, Zhao et al. [36], developed thermosensitive liposomes co‐loaded with a photosensitive dye and tobramycin. Upon NIR irradiation, they showed a temperature increase to 45°C, which triggered drug release and enhanced antibacterial efficacy against *P. aeruginosa* by twofold. With a different approach, Yuan et al. [78], combined PTT with nitric oxide (NO) release, reducing S. *aureus* biofilm mass by 5‐times. With a similar approach, they also obtain the remote eradication of *S. aureus* biofilm on titanium implants in vivo [79]. Here, CoPc‐Lig NPs reduced the viability of *S. aureus* and *P. aeruginos*a biofilms by 40% and 30% respectively, after treatment with NIR, albeit transiently. Indeed, we observed biofilm recovery after 24 h from treatment (Figure S6), suggesting that complete eradication may require repeated treatments, also in combination with local delivery of antibiotics. The stability of the NPs signal observed in the PA tests confirms the capability of this system to sustain repeated irradiations. Furthermore, the in vivo tests—albeit in a simplified wound model in larvae, indicated the safety of the photothermal approach, further supporting its applicability. This result should be further confirmed in well‐established murine wound models.

Another interesting aspect of this work is the exploration of PA imaging for real‐time monitoring of the biofilm, a fundamental aspect to adjust therapies to the dynamic nature of CWs. PA imaging exploits the capacity of electromagnetic energy to generate acoustic waves [80, 81]. This technique allows real‐time localization of chromophores in deep tissue with high spatial and temporal resolution, without using ionizing radiation [82]. In our study, CoPc‐Lig NPs were successfully detected in ex vivo tissue phantoms and within *S. aureus* biofilms, showing a stable signal over time under continuous irradiation, suggesting the potential for long‐term monitoring after *in situ* application (Figures 6 and 9; Figure S4). Systems allowing continuous wound monitoring are highly sought. To this aim, smart dressings incorporating pH or glucose sensing capacity using visual cues or integrated electronics have been proposed [41, 42, 83, 84]. For instance, Mirani et al. [41], developed a hydrogel dressing that changed colour in response to pH, an important indicator of bacterial presence, while Zhu et al. [42], engineered a zwitterionic hydrogel capable of sensing both pH and glucose. Other approaches integrate sensors into patches to monitor wound conditions and to trigger the on‐demand delivery of therapeutic compounds [43, 44]. So far, PA imaging has not been broadly investigated for monitoring CWs. However, it has great potential in the evaluation of wound progression in real time, given its capacity to visualize the wound tissue at higher penetration depth compared to traditional optical imaging [85]. Wound heterogeneity may represent a challenge, since extensive granulation or necrotic tissue could influence depth penetration, but this point strictly depends on the optical window and laser fluence used [86]. Moreover, PA imaging is expected to be independent from exudate quantity and composition, since exudates do not present strong absorption optical properties in the NIR [86]. Hariri et al. [87], explored the use of PA imaging to monitor the wound formation of pressure ulcers in murine models, showing the ability of this technique to differentiate among wound stages. Our results show that PA monitoring is feasible with CoPc‐Lig NPs, warranting their further investigation as multifunctional theragnostic agents in the treatment and monitoring of CWs. Further experiments will focus on testing PA imaging in rodent wound models to assess its suitability for analysing wounds at different stages, depths, and exudative states.

## 4 Conclusions

In this work, CoPc was combined with a lignin‐based material to obtain CoPc‐Lig NPs with small size, round‐shaped morphology, anti‐inflammatory properties, and cytocompatibility at high concentrations. The NPs were easily internalized into bacterial biofilms and showed thermal increment below 50°C when excited with NIR light. These temperatures are recognized to enhance tissue regeneration and augment the antibacterial efficacy of different therapeutic agents [36, 37]. Notably, following NIR irradiation, a significant decrease in bacterial viability and disruption of biofilm structure were observed. Future tests will focus on optimizing the photothermal treatment conditions to potentiate the antibiofilm action. With the same aim, the combination of photothermal treatment with debridement and other antibacterial agents will be investigated. The synergic anti‐inflammatory and anti‐biofilm properties of CoPc‐Lig NPs make them a promising and innovative treatment for CWs. Moreover, CoPc‐Lig NPs displayed PA behavior, which may allow long‐term wound monitoring, as demonstrated in ex vivo tissue phantoms and within *S. aureus* biofilms, opening the road for future applications of these NPs in the theragnostic field.

## 5 Materials and Methods

### 5.1 Materials and Reagents

Protobind 6000 sulfur‐free Lig powder was acquired from Green Value (Switzerland). Gallic acid, tannic acid (TA), 3′,5′‐dimethoxy‐4′hydroxyacetophenone (97%) (acetosyringone), guaiacol, 2,2′‐azino‐bis(3‐ethylbenzothiazoline‐6‐sulfonic acid) diammonium salt (ABTS), phosphate‐buffered saline (PBS), Folin–Ciocalteu phenol reagent, sodium carbonate, dimethyl sulfoxide (DMSO), Mueller‐Hinton Broth (MHB), Brain‐Heart Infusion (BHI), D‐glucose, agar, Tryptic Soy Broth (TSB), paraformaldehyde (PFA 4%), and Dulbecco's modified Eagle's medium (DMEM) were obtained from Sigma‐Aldrich (Madrid, Spain and Milan, Italy). Alpha‐cyclodextrins (αCDs) were purchased from TCI Chemicals Europe (Zwijndrecht, Belgium). CellTiter 96 AQueous One Solution Cell Proliferation Assay (MTS) was purchased from Promega Corporation (Milan, Italy). EnzChek Gelatinase/Collagenase Assay Kit was supplied by Invitrogen, Life Technologies Corporation (Madrid, Italy). Cobalt‐phthalocyanine (CoPc), 4′,6‐diamidino‐2‐phenylindole (DAPI), and Alexa Fluor 594 phalloidin were obtained from ThermoFisher Scientific (Milan, Italy). Novozym 51003 laccase from Miceliophtora termophila was provided by Novozymes (Bagsvaerd, Denmark). The laccase exhibited an enzymatic activity of 179 U/mL, defined as the quantity of enzyme needed to convert 1 µmol of ABTS to its cation radical (ε436 = 29,300 M^−1^ cm^−1^) in 5 mm Britton Robinson buffer (pH 5) at 40°C. Bacterial strains (*Staphylococcus aureus* ATCC 6538 and *Pseudomonas aeruginosa* ATCC 9027) and human fibroblast cell line (ATCC‐SCRC‐1041, HFF‐1) were purchased from the American Type Culture Collection (ATCC LGC Standards, Italy). Human keratinocytes (HaCaT) were purchased from “Istituto Zooprofilattico Sperimentale della Lombardia e dell'Emilia Romagna Bruno Ubertini” (Italy). The water utilized throughout the experiments was purified via the Milli‐Q plus system (Millipore).

### 5.2 Enzymatic Functionalization of Lignin

Lig was functionalized with TA by modifying a previously reported procedure [88]. Briefly, Lig (10 mg/mL) was soaked in sodium acetate buffer (50 mm, pH 5, 50°C) containing acetosyringone, one of the most efficient natural enhancers in laccase‐mediated oxidation [89], at a concentration of 1.5 mg/mL. Subsequently, laccase (1% v/v) was introduced into the Lig solution under stirring for 1 h at 50°C to initiate the oxidative reaction. Finally, TA (10 mg/mL) was incorporated into the solution, and the reaction continued under stirring for 2 h at 50°C. Unreacted TA was separated from the resulting material by centrifugation at 4750 rpm at 4°C for 15 min. The resulting powder, designated as Lig‐TA, was then freeze‐dried and stored for further characterization.

#### 5.2.1 ATR‐FTIR Spectroscopy

Successful conjugation of TA to Lig was investigated with attenuated total reflectance–Fourier transform infrared (ATR‐FTIR) spectroscopy. ATR‐FTIR analysis was conducted on Lig‐TA using a PerkinElmer Spectrum 100 FTIR spectrometer (PerkinElmer, Waltham, MA, USA). Spectra were generated by averaging 32 scans over the spectral range from 4000 to 600 cm^−1^ at a resolution of 1 cm^−1^. All analyses were carried out at room temperature. Spectra of Lig and TA were also obtained for comparison.

#### 5.2.2 Measurement of the Phenolic Content

To confirm the presence of TA, variations in the phenolic content of Lig‐TA were determined spectrophotometrically using Folin–Ciocalteu reagent. Briefly, 20 µL of a water solution containing Lig‐TA (1 mg/mL) was combined with 100 µL of 20% w/v sodium carbonate (Na2CO3) and 80 µL of 0.2 N Folin–Ciocalteu phenol reagent. The resulting mixture was incubated for 10 min in the dark. The absorbance at 765 nm was then measured using an Infinite M200 spectrophotometer (TECAN). Gallic acid (GA) was used to obtain a calibration curve (0.1–1 mg/mL). All samples were measured in triplicate, and the results were expressed in GA equivalents (GAE) per g of material.

### 5.3 Preparation and Characterization of CoPc‐Lig NPs

#### 5.3.1 Preparation and Characterization of CoPc‐Lig NPs

CoPc‐Lig NPs were obtained by mixing CoPc with Lig‐TA, since the lignin‐based material acts as a dispersant of CoPc [49]. Briefly, CoPc at 7.85 mg/mL was suspended in DMSO through sonication for 2 min, while Lig‐TA was dissolved at 5 mg/mL in deionized water at pH 8.5. The two solutions were mixed at 60°C at a Lig‐TA/CoPc mass ratio of 3:2 (determined after testing different conditions, as reported in the Table S1) and stirred at 500 rpm for 2 h. To purify the samples from unreacted Lig‐TA, centrifugation at 17 000 rpm for 20 min was performed. The obtained pellet was resuspended in MilliQ water and sonicated for 30 s. A second centrifugation step was performed at 1200 rpm for 5 min to remove large CoPc‐Lig particle aggregates. The waste products of the centrifugation steps were collected and analyzed separately by ATR‐FTIR and UV–vis spectroscopy (Varioskan LUX Multimode Microplate Reader, Life Technologies, Carlsbad, California, USA) to evaluate their composition (see Figures S1–S3), as detailed above. The yield of the NPs production process was calculated on the freeze‐dried products by using Equation (1):

(1) $$\mathrm{Production}\;\mathrm{Yield}=\frac{M_{\mathrm{NPs}}}{M_{\mathrm{tot}}}\;100$$

where *M_NPs_* is the weight of the CoPc‐Lig NPs, measured after freeze‐drying, and *M_tot_* is the total weight of product obtained before purification.

Cobalt in CoPc‐Lig NP was quantified using Inductively Coupled Plasma Mass Spectrometry (ICP‐MS). Briefly, samples were digested with 20% (v/v) HNO_3_ at 100°C for 3 h, then diluted to a final concentration of 2% v/v and filtered through a 0.2 µm filter. Quantification of cobalt within the CoPc‐Lig NPs was performed via ICP‐MS (ICP‐MS 7800, Agilent Technologies, Santa Clara, CA, USA), calibrated using an internal standard (45Rh) and a standard curve of ^59^Co.

The size, polydispersity index (PDI), and surface charge (ζ potential) of CoPc‐Lig NPs were characterized through Dynamic Light Scattering (DLS, Litesizer 500 from Anton Paar, Turin, Italy), after resuspending NPs in water. The morphology of CoPc‐Lig NPs was assessed by transmission electron microscopy (TEM) (TALOS F200X, Thermo Scientific). To confirm the presence of cobalt and its distribution within the NPs matrix, Energy Dispersive X‐ray Spectrometry (EDX) analysis was coupled with TEM imaging.

#### 5.3.2 Cytocompatibility and Cell Internalization of CoPc‐Lig NPs

The cytocompatibility of CoPc‐Lig NPs was assessed on fibroblasts (HFF‐1) and keratinocytes (HaCaT). Briefly, cells were seeded in a 96‐well tissue‐culture‐treated polystyrene plate at a density of 10 000 cells per well in 100 µL of DMEM supplemented with 200 mm L‐glutamine, 1% penicillin/streptomycin, and 10% (v/v) fetal bovine serum (FBS). After 24 h of incubation (37°C, 5% CO_2_), CoPc‐Lig NPs were dispersed in cell culture medium and added to the wells containing the cells at concentrations ranging from 0.1 mg/mL to 4 mg/mL. After 24 h of incubation, cell viability was assessed using the CellTiter 96 AQueous One Solution Cell Proliferation Assay (MTS). Briefly, the medium was replaced with 100 µL of fresh medium, and 20 µL of MTS reagent was added. After 1 h of incubation, the absorbance was measured at 490 nm. Cells cultured without NPs served as a control (representing 100% cell viability).

The internalization of CoPc‐Lig NPs into fibroblasts and keratinocytes was assessed qualitatively through fluorescence microscopy using a spinning disk confocal microscope (Nikon ECLIPSE Ti2‐E). Briefly, cells were seeded at 70 000 cells per well in a 48‐well plate on glass sides and incubated at 37°C. After 24 h, CoPc‐Lig NPs were added at 0.5 mg/mL and incubated for 24 h. Then, the cells were fixed for 20 min with 4% PFA, permeabilized with TritonX 0.5% for 10 min, and stained with DAPI to mark cell nuclei and Alexa Fluor 594‐phalloidin to mark the cytoskeleton. Brightfield and fluorescence images were acquired.

#### 5.3.3 Antioxidant Activity of CoPc‐Lig NPs

The ROS scavenging capacity of CoPc‐Lig NPs was assessed through the 2,2‐diphenyl‐1‐picrylhydrazyl (DPPH) assay. Briefly, 25 µL of sample suspension (0.1, 0.2, 0.3, 0.4, 0.5, 0.6, and 1.2 mg/mL in MilliQ water) and 75 µL of DPPH solution (0.1 mm in ethanol) were mixed and incubated for 30 min at room temperature. After incubation, the samples were centrifuged at 10000 rpm for 5 min to eliminate the suspended materials, and the absorbance at 517 nm was measured. Samples mixed with ethanol were used as background, while DPPH mixed with MilliQ water represented the 100% DPPH signal.

#### 5.3.4 MMPs Inhibition Assay

The MMP inhibition capacity of CoPc‐Lig NPs was determined using the EnzCheck Gelatinase/Collagenase Assay Kit, with some modifications. Initially, CoPc‐Lig NPs at different concentrations (0.1, 0.2, 0.4, and 0.8 mg/mL) were dispersed in 500 µL of reaction buffer (0.05 m Tris HCl, 0.15 m NaCl, 5 mm CaCl_2_, 0.2 mm sodium azide, pH 7.6). Subsequently, 5 µL of 500 U/mL collagenase type IV from Clostridium histolyticum was added to achieve a final concentration of 5 U/mL. The solution was then incubated at 37°C for 24 h. After incubation, 100 µL of the sample was mixed with 80 µL of reaction buffer and 20 µL of fluorescein‐conjugated gelatine (100 µg/mL in Milli‐Q water). The plate was incubated for 30 min in the dark at room temperature, and fluorescence was measured at λ_ex/em_ = 493/528 nm. Collagenase activity was determined as a percentage of enzyme inhibition compared to the control (a reaction mixture containing the enzyme and the substrate but without NPs).

#### 5.3.5 Antibacterial and Antibiofilm Activity of CoPc‐Lig NPs

The antibacterial activity of CoPc‐Lig NPs was determined on *S. aureus* and *P. aeruginosa* through the viable cell counting method to calculate the colony‐forming units per mL (CFU/mL). Briefly, bacterial suspensions (5 × 10^5^ CFU/mL) in MHB were incubated at 37°C with CoPc‐Lig NPs at different concentrations (0.125, 0.25, 0.5, 1, 2, 4 mg/mL). After 24 h of incubation, treated and untreated bacteria were serially diluted in PBS and plated onto agar plates to count the colony‐forming units. The test was performed in triplicate, and controls with untreated bacteria were considered as a reference. The reduction of bacterial growth was calculated as indicated in Equation (2):

(2) Logreductionofbacterialgrowth=log(Ct)−log(Cc)

where *C_t_* and *C_c_* are the concentrations of CFU/mL for the treated and untreated bacteria, respectively. The concentration of CFU was calculated as:

(3) $$C_{t}=\frac{{n_{t}}^{*}10^{\wedge }d}{v}$$

where *n_t_* is the number of counted colonies, *d* is the dilution factor, and *v* is the volume of the bacterial suspension.

The antibiofilm activity of CoPc‐Lig NPs *per se* (i.e., without NIR irradiation) was determined on the same *S. aureus* and *P. aeruginosa* strains as follows. *S. aureus* and *P. aeruginosa* suspensions were prepared in TSB and BHI, respectively, and seeded at 10^7^ CFU/mL. After 24 h of incubation, the obtained biofilms were washed with PBS, and a suspension of CoPc‐Lig NPs at different concentrations (0.125, 0.25, 0.5, 1, 2, 4 mg/mL) in the appropriate medium was added. After an additional 24 h incubation period at 37°C, the supernatant was removed, the biofilms were washed once with PBS, and bacterial viability was assessed through CellTiter 96 assay (Promega, Milan, Italy). Briefly, 100 µL of bacterial medium (TSB for *S. aureus* and BHI for *P. aeruginosa*) was mixed with 20 µL of the CellTiter reagent, added to the biofilm, and incubated for 30 min at 37°C. The medium was collected, and the absorbance was measured at 490 nm.

#### 5.3.6 Internalization of CoPc‐Lig NPs into *S. aureus* and *P. aeruginosa* Biofilms

Scanning Electron Microscope (SEM) imaging was performed to verify the internalization of CoPc‐Lig NPs in biofilms. Briefly, *S. aureus* and *P. aeruginosa* biofilms were formed on silicon wafers as follows. *S. aureus* and *P. aeruginosa* suspensions were prepared in TSB and BHI, respectively, and seeded at 10^7^ CFU/mL on the wafers. After 24 h of incubation, the biofilms were washed with PBS, and a suspension of CoPc‐Lig NPs at 0.5 mg/mL in the appropriate medium was added. After an additional incubation of 24 h at 37°C, the supernatant was removed, and the biofilms were washed once with PBS and fixed for 1 h in a 4% PFA solution. Subsequently, biofilms were dehydrated by incubation with increasing concentrations of ethanol (25%, 50%, 75%, and 100%) for 1 h each. The samples were subjected to SEM analysis and Energy Dispersive X‐Ray Analysis (EDX). Untreated bacterial biofilms were used as controls.

The internalization of CoPc‐Lig NPs into *S. aureus* biofilms was quantified through UV–vis spectroscopy. Briefly, *S. aureus* biofilms were formed as described above and incubated with CoPc‐Lig NPs at different concentrations (0.1–0.5 mg/mL). After 24 h, the biofilm was lysed with DMSO/0.1 m glycine buffer (pH 10.2) solution (7:1), and the absorbance at 620 nm was measured to quantify the CoPc‐lig NPs internalized within the biofilm. The % of internalized NPs was measured as:

(4) $$\%\;\mathrm{internalised}\;\mathrm{NPs}=\frac{\mathrm{quantity}\;\mathrm{of}\;\mathrm{NPs}\;\mathrm{internalised}}{\mathrm{initial}\;\mathrm{quantity}\;\mathrm{of}\;\mathrm{NPs}\;\mathrm{added}\;\mathrm{to}\;\mathrm{the}\;\mathrm{biofilm}}100$$

#### 5.3.7 Photoacoustic Response of CoPc‐Lig NPs

PA spectra of CoPc‐Lig NPs were acquired with multimodal imaging platform Vevo LAZR‐X (Fujifilm VisualSonics Inc.), which produces real‐time PA images co‐registered with B‐mode images. PA characterization of CoPc‐Lig NPs was performed by placing the NPs in a custom‐made phantom, comprising a plastic box with a series of coplanar micro‐medical polyethylene (PE) tubes with an internal diameter (ID) of 580 µm and an external diameter (ED) of 990 µm that are non‐radiopaque, non‐reactive, and do not contain plasticizers. The CoPc‐Lig NPs at 0.2 mg/mL were loaded into PE tubes (∼40 µL each tube). A tube loaded with water was used as a background. The generation of the PA effect on the samples was stimulated with a frequency of 20 Hz by 10 ns laser pulses with a mean energy of around 50 mJ within a wavelength range of 680–970 nm. The photoacoustic and ultrasound imaging (PAUS) probe was the MX550, 40 MHz central band, and provided an axial resolution of 60 µm. The acquisition time for the absorption spectrum was ∼40 s per 59 wavelengths (at 5 nm step). The PA values were measured in regions of interest (ROIs), which were selected around the section of each tube as reported in [90, 91].

The PA detectability of CoPc‐Lig NPs was measured in ex vivo phantoms, composed of bovine muscular tissues. These phantoms were injected with NPs at 0.2 mg/mL and analyzed with the PA probe. The PA values were measured in two ROIs of the same dimension, the first around the NPs injection zone and the second in an NPs‐free area. From these data, the signal‐to‐noise ratio (SNR), the contrast‐to‐noise ratio (CNR), and the Coefficient of Variation (CV%) were calculated as follows:

(5) $$\mathrm{SNR}=\frac{\mathrm{signa}l_{\mathrm{NPs}}\;}{\sigma_{\mathrm{NPs}}}$$

(6) $$\mathrm{CNR}=\frac{\mathrm{signa}l_{\mathrm{NPs}}\;-\;\mathrm{signa}l_{\mathrm{No}\;\mathrm{NPs}\;}\;}{\sqrt{\sigma_{\mathrm{NPs}}^{2}\;-\;\sigma_{\mathrm{No}\;\mathrm{NPs}}^{2}\;}}$$

(7) $$\mathrm{CV}\%=\frac{1}{\mathrm{SNR}}100$$

where *signal_NPs_* is the average signal of the ROI in the injection zone, *signal*_*No* *NPs*_ is the average signal of the ROI in the NPs‐free area, and σ_*NPs*_ and σ_*No* *NPs*_ are their respective standard deviations.

The PA activity of CoPc‐Lig NPs within *S. aureus* biofilm was also measured. The bacteria biofilms were grown on 0.5 cm‐thick custom‐made polydimethylsiloxane (PDMS; Sylgard 184, Dow Chemical, Midland, USA) slides. After incubation with CoPc‐Lig NPs at 0.5 mg/mL for 24 h, the biofilms were washed with PBS and fixed for 1 h with 4% PFA. To facilitate imaging with the ultrasound probe, an additional PDMS layer was poured on the fixed biofilms, and the samples were incubated for 48 h at room temperature before analysis. For each ROI, the photostability (PHSt) and PA spectral trend were analyzed. The PA spectrum was evaluated from 680 nm to 970 nm. The PHSt was evaluated under pulsed irradiation at 710 nm for over 1 min.

#### 5.3.8 Photothermal Activity of CoPc‐Lig NPs

For testing the photothermal activity of CoPc‐Lig NPs within *S. aureus* biofilm, the bacterial biofilms were grown on 0.5 cm‐thick custom‐made polydimethylsiloxane (PDMS; Sylgard 184, Dow Chemical, Midland, USA) slides. To form the PDMS slides, a PDMS solution was prepared by mixing the polymeric base with a curing agent at 10:1 weight ratio. The solution was poured into 6‐well plate molds and incubated for 48 h to allow the complete cross‐linking of PDMS. *S. aureus* suspension at 10^7^ CFU/mL was seeded on the PDMS slides. After 24 h of incubation, the slides were washed with PBS to remove non‐adherent bacteria and moved to a new well plate, where the CoPc‐Lig NPs were added at a concentration of 0.5 mg/mL. After 24 h of incubation, the biofilms were washed again with PBS and fixed for 1 h with PFA 4% before imaging. Near Infrared Thermal Imaging was performed by illuminating biofilm samples through continuous wavelength laser illumination at 660 nm at room temperature under controlled humidity, laser beam light, and power distribution. The biofilm samples were illuminated by the laser for 120 s, while recording the thermal gradient with a near‐infrared camera (FLIR T560). The datasets were collected in post‐processing by using the FRS Software (FLIR) and fitted by the curve fitting tool in MATLAB.

#### 5.3.9 Evaluation of Anti‐Biofilm Activity under NIR Irradiation

To test anti‐biofilm activity after NIR irradiation, *S. aureus* and *P. aeruginosa* biofilms were treated for 24 h with CoPc‐Lig NPs at 4 mg/mL. Then, the biofilm plates were placed on a heater to maintain the basal temperature at 37°C and irradiated for 15 min at 660 nm. The bacteria's viability was assessed by CellTiter 96 assay immediately and 24 h after NIR treatment. The biofilm integrity was analyzed by SEM imaging, by preparing biofilm samples on wafer slides, and treating them as described above. The biofilm structure before and after NIR treatment was observed under a confocal microscope. For this purpose, biofilms were grown on glass slides following the same preparation protocol used for SEM imaging and incubated for 24 h with CoPc‐Lig NPs (4 mg/mL) to allow internalization. After treatment with NIR light, the supernatant was removed, biofilms were fixed for 1 h in a 4% PFA solution, and bacterial DNA was stained with DAPI. After washing with PBS, the samples were analyzed using a spinning disk confocal microscope.

### 5.4 Preliminary In Vivo Validation of CoPc‐Lig NPs on a Wound Model in *Galleria mellonella*

*Galleria mellonella* larvae were purchased from a local fishing supply store and maintained at room temperature until use. For each experimental group, five healthy larvae were selected, each with an average weight of (250 ± 50) mg. Before experimentation, larvae were starved for 24 h to minimize variability associated with recent feeding. To reduce potential contamination, larvae were surface sterilized with 70% ethanol and subsequently transferred to sterile Petri dishes.

**FIGURE 10:**
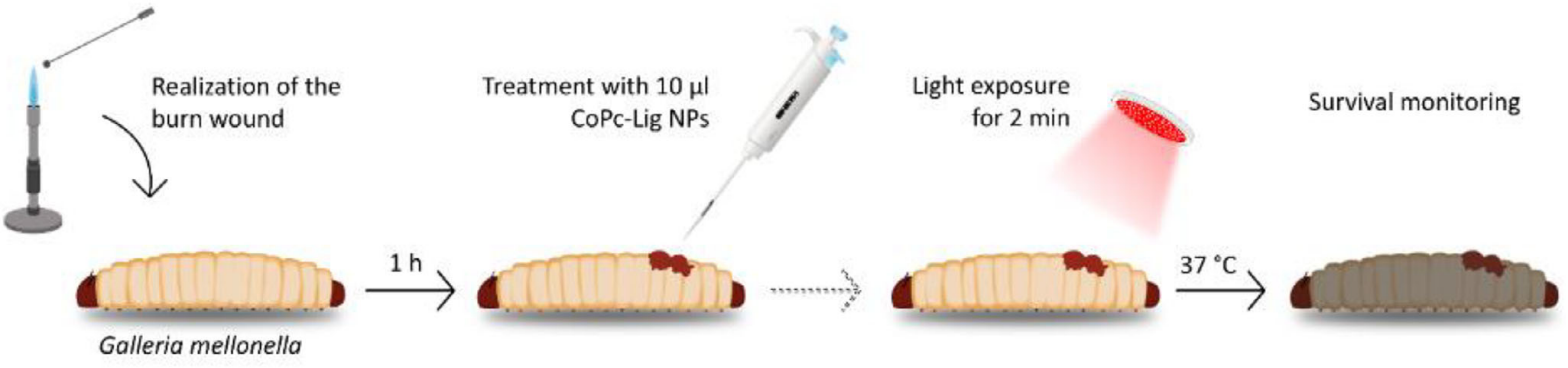
Schematic representation of the protocol used to establish the wound model in *Galleria mellonella* larvae. Illustration created using Inkscape.

The wound was induced using a previously published protocol [92, 93, 94] and is shown in Figure 10. The safety of CoPc‐Lig NPs was assessed on larvae with intact skin before and after irradiation with 660 nm‐radiation (Groups “CoPc‐Lig NPs” and “CoPc‐Lig NPs+Lamp”, respectively). Untreated larvae served as the control group (Group CTRL).

For the wound model, a metal spatula was heated and applied to the dorsal mid‐section of each larva for 4 s, generating a burn area of approximately 2 mm^2^ (“wound” group). Larvae exhibiting haemolymph leakage or protruding fat body were excluded and euthanized by placement at −20°C. One hour after burn induction, larvae were treated with 10 µL of CoPc‐Lig NPs (1 mg/mL) (Group “Wound+CoPc‐Lig NPs”). Immediately after the NPs application, larvae were exposed to 660 nm irradiation for 2 min (Group “Wound+CoPc‐Lig NPs+Lamp”). Following treatment, larvae were incubated at 37°C, and mortality was monitored, with death defined by complete melanization and/or total loss of motility.

### 5.5 Statistical Analysis

Results are reported as mean ± standard deviation. Statistical analysis was performed through Microsoft Excel for Windows 10. To compare results, one‐way ANOVA analyses were performed, followed by Bonferroni's multiple comparison test. Statistical significance was assessed as follows: n.d.—non‐significant difference, ^*^ *p* < 0.05, ^**^ *p* < 0.01, and ^***^ *p* < 0.001.

*G. mellonella* survival profiles were visualized using the Kaplan‐Meier survival curves. To assess statistical differences between survival curves, Pairwise Log‐Rank tests were performed for all group combinations using the logrank‐test function of lifelines in Python 3.

## Funding

The Ministry of University and Research (MUR) Facilitated This Funding Via Call No. 3277, tagged to Project Code ECS_00000017, and as per MUR Directoral Decree No. 1055 issued on June 23, 2022. This research was supported by the European Union ‐ NextGenerationEU, under the Innovation Ecosystem project ‘THE ‐ Tuscany Health Ecosystem’ (Project Code: ECS00000017, CUP: B83C22003930001), Spoke 1. Additionally, this work was carried out within the framework of the PNRR project ‘SEE‐LIFE’ (StrEngthEning the ItaLIan InFrastructure of Euro‐BioImaging, CUP: B83C22003930001. This work was supported by the European Union – Next Generation EU, Mission 4 – Component 2, through the 3DPathoSkin project (PE_00000019 – HEAL ITALIA; CUP E93C22001860006). Additional support for S.V. was provided by the Italian Ministry of Research under Ministerial Decree No. 351/2022 – PNRR Mission 4, Component 1.

## Conflicts of Interest

The authors declare no conflict of interest.

## Supporting information




**Supporting File**: mabi70133‐sup‐0001‐SuppMat.pdf.

## Data Availability

The data that support the findings of this study are available from the corresponding author upon reasonable request.
