# Supplementary material for: Lignin Nanoparticles Containing Cobalt‐Cyanine Complexes: Potential Multifunctional Platforms for Photoacoustic Imaging and Photothermal Treatment of Bacterial Biofilms in Chronic Wounds
Source: Macromol Biosci. 2026 Feb 3;26(2):e00532. doi: 10.1002/mabi.202500532 (PMC12868942; doi:10.1002/mabi.202500532)
Supplement: Supplementary file 1 — Supporting File: mabi70133‐sup‐0001‐SuppMat.pdf. [file MABI-26-e00532-s001.pdf]

Development of multifunctional CoPc-Lig nanoparticles for PA Imaging and photothermal treatment of typical chronic wound bacterial biofilms

Giulia Crivello[1,,†], Matteo Felice Pezzuto[1,†], Paolo Armanetti[2,†], Claudio Cassino[3], Chiara Ceresa[4], Letizia Fracchia[4], Claudia Catarinicchia[2], Stefania Villani[5], Pietro Alifano[6], Christian Demitri[6], Luca Menichetti[2], Tzanko Tzanov[7], Gianluca Ciardelli[1], and Clara Mattu[1]*

[1] Department of Mechanical and Aerospace Engineering, Politecnico di Torino, Corso Duca degli Abruzzi 24, 10129, Torino, Italy; G.C. giulia.crivello@polito.it; M.F.P. matteo.pezzuto@polito.it; Gi.C. gianluca.ciardelli@polito.it; C.M. clara.mattu@polito.it;

[2] Institute of Clinical Physiology, National Research Council, Via Giuseppe Moruzzi 1, 56124, Pisa, Italy; P.A. paolo.armanetti@cnr.it; C.Cl. claudiacatarinicchia@cnr.it; L.M. luca.menichetti@cnr.it;

[3] Department of Science and Technological Innovation, Università del Piemonte Orientale "A. Avogadro", Viale Teresa Michel 11, 15121 Alessandria, Italy; Cl.C. claudio.cassino@uniupo.it;

[4] Department of Pharmaceutical Sciences, Università del Piemonte Orientale "A. Avogadro", Largo Donegani 2, 28100, Novara, Italy; C.C. chiara.ceresa@uniupo.it; L.F. Letizia.fracchia@uniupo.it;

[5] Department of Engineering for Innovation, University of Salento, Via per Monteroni, 73100, Lecce, Italy; S.V. stefania.villani@unisalento.it

[6] Department of Experimental Medicine, University of Salento, Via per Monteroni, 73100, Lecce, Italy; Pi. A. pietro.alifano@unisalento.it; C.D. christian.demitri@unisalento.it

[7] Group of Molecular and Industrial Biotechnology, Department of Chemical Engineering, Universitat Politècnica de Catalunya, 08222 Terrassa, Spain; T.T. tzanko.tzanov@upc.edu.

*** Corresponding author:** giulia.crivello@polito.it;

† Equal contribution/Shared first authorship

Supplementary materials

Table S1. Summary of the CoPc-Lig NPs produced. CoPc-Lig NPs were synthesized by combining CoPc and Lig-TA at different mass ratios. The obtained NPs presented a hydrodynamic diameter between 220 and 250 nm, PDI between 0.14 and 0.24, ζ Potential between -38 and -28 mV, and a production yield between 3.5 and 6.5%. To minimize the PDI and maximise the production yield,

NPs obtained at 3:2 Lig-TA/CoPc weight ratio (highlighted in red) were selected, referred to as CoPc-Lig NPs in the main paper.

NP name	Lig-TA/CoPc mass ratio	Hydrodynamic diameter (nm)	PDI	ζ Potential (mV)	Production Yield (%)
**CoPc-Lig_2:5**	2:5	223 ± 1	0.22 ± 0.03	-30.5 ± 0.5	4.5
**CoPc-Lig_2:3**	2:3	250 ± 3	0.24 ± 0.01	-35.8 ± 0.5	6
**CoPc-Lig_1:1**	1:1	222 ± 5	0.21 ± 0.03	-37.9 ± 0.5	3.5
**CoPc-Lig NPs**	3:2	247 ± 1	0.14 ± 0.03	-31.7 ± 0.5	6.5
**CoPc-Lig_5:2**	5:2	232 ± 1	0.19 ± 0.03	-28.9 ± 0.2	4
**CoPc-Lig_5:1**	5:1	231 ± 6	0.21 ± 0.03	-30.2 ± 0.2	6

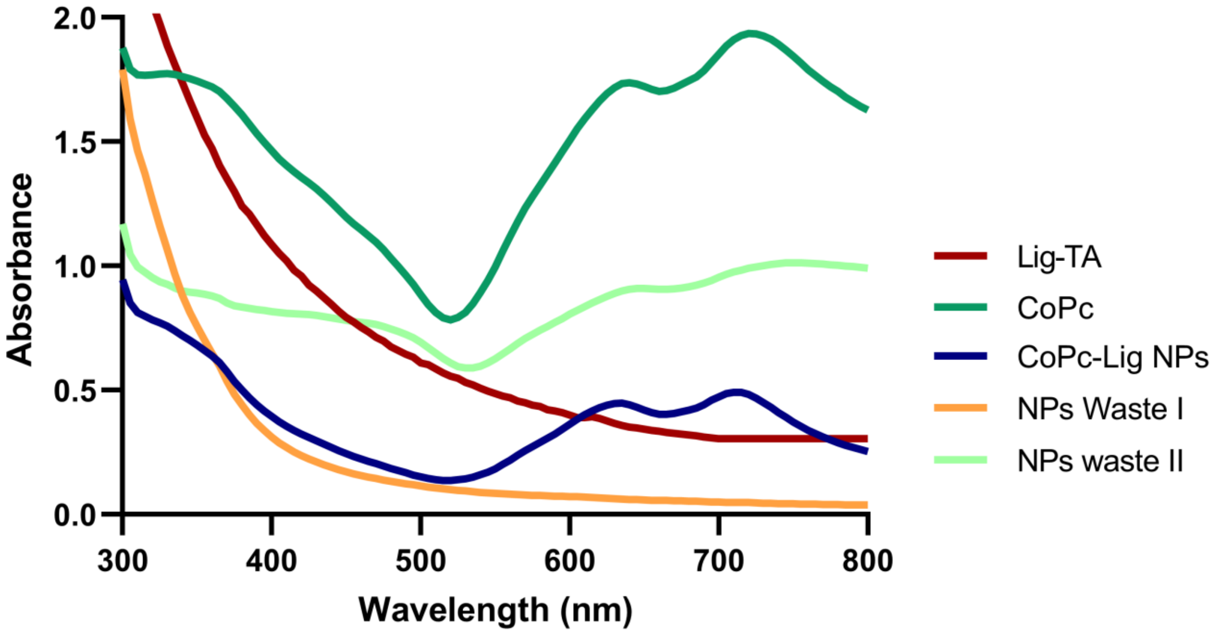

Figure S1. UV-vis spectra of CoPc-Lig NPs compared to CoPc, Lig-TA, and the waste collected after the two centrifugation steps. UV-vis spectrum of CoPc-Lig NPs (blue) indicated two adsorption peaks at 630 nm and 710 nm, characteristic of CoPc (dark green), therefore confirming that CoPc was present in the NPs. A similar spectrum profile was detected for NPs waste II (light green), which was collected during the second washing step at low centrifugation speed. On the other hand, in the sample NPs-waste I (orange), no peaks indicative of CoPc were found, and the spectrum was more similar to that of Lig-TA (red). These results indicated that the first centrifugation step mainly eliminated the unreacted Lig-TA, while unreacted CoPc or large CoPc-Lig NPs aggregates were removed during the second centrifugation step.

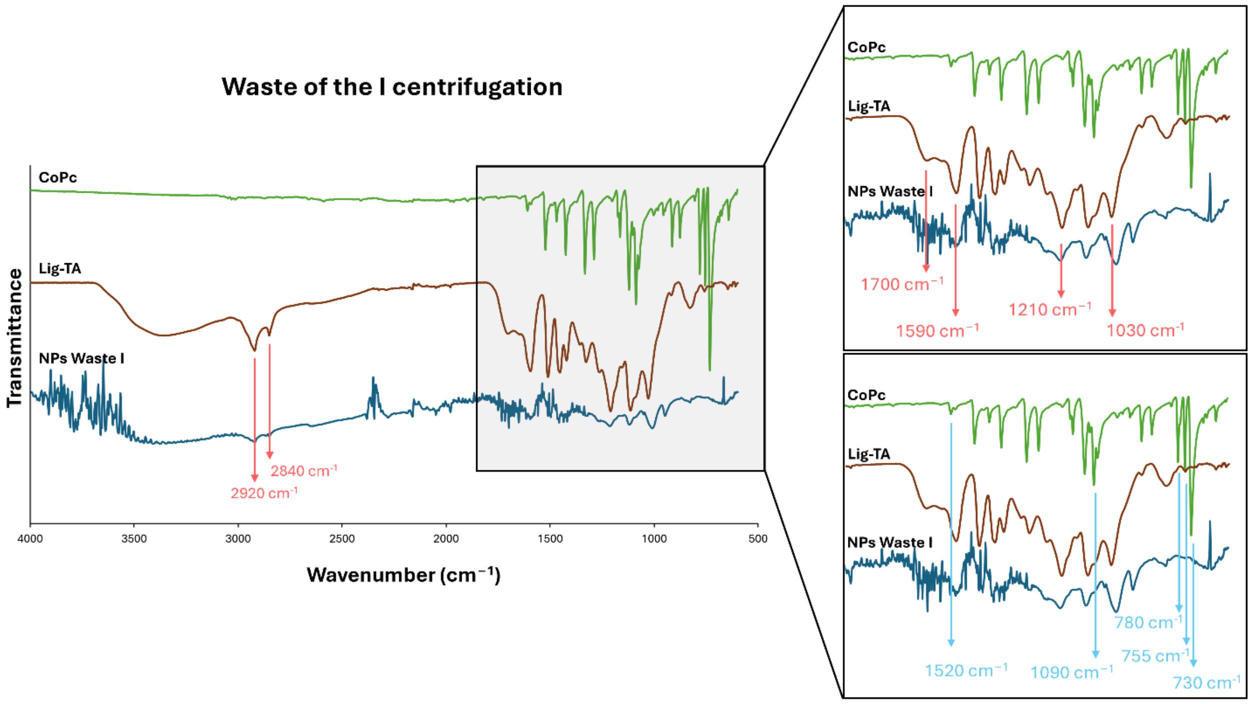

Figure S2. ATR-FTIR spectra of the waste collected after the first centrifugation step in comparison to the spectrum of bare Lig-TA and CoPc. Red arrows indicate Lig-TA-related peaks, while blue arrows indicate CoPc-related peaks. In the waste of the first centrifugation, only peaks related to Lig-TA were observed, indicating the presence of unreacted Lig-TA.

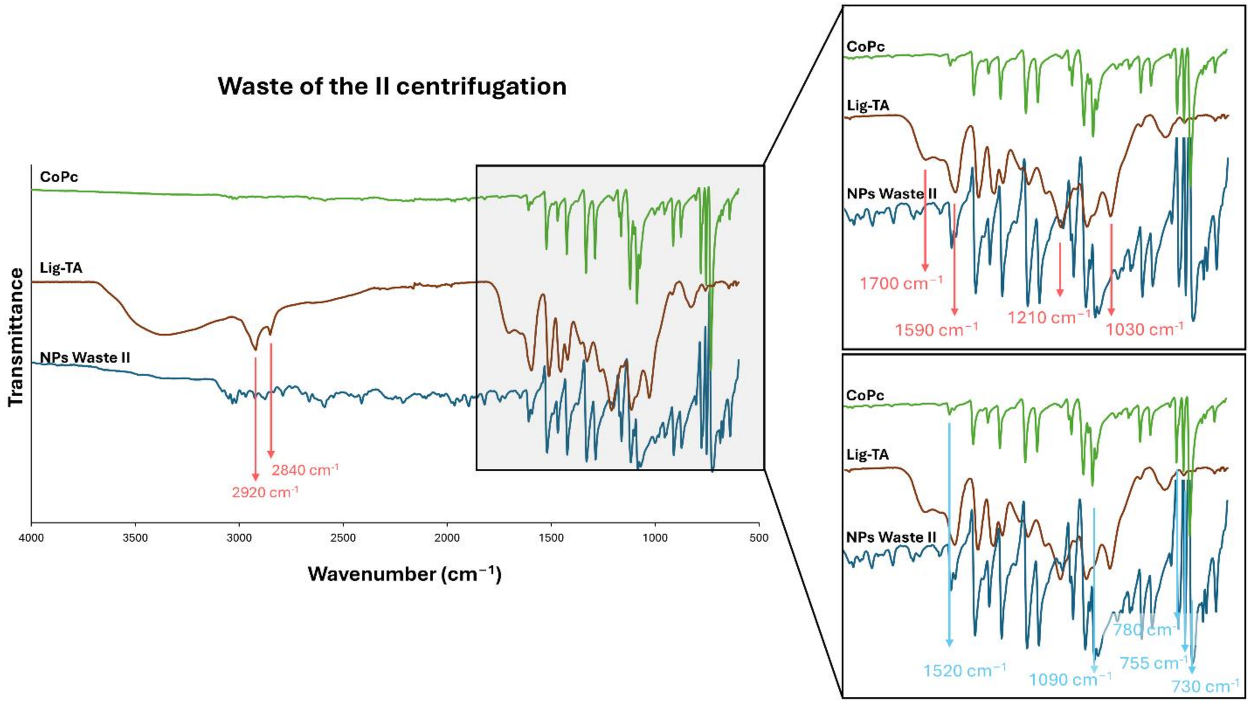

Figure S3. ATR-FTIR spectra of the waste collected after the second centrifugation step in comparison to the spectrum of bare Lig-TA and CoPc. Red arrows indicate Lig-TA-related peaks, while blue arrows indicate CoPc-related peaks. In the waste of the first centrifugation, only peaks related to CoPc were observed, indicating the presence of unreacted CoPc or large CoPc NPs aggregates.

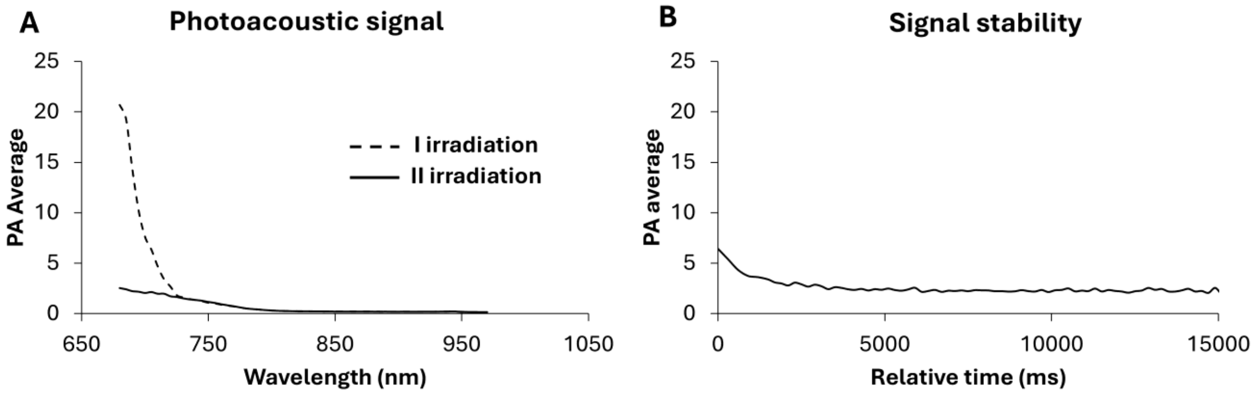

Figure S4. PA characterization of CoPc-Lig NPs in aqueous suspension. (A) PA spectra of excitation at different wavelengths measured initially (I irradiation) and after prolonged pulsed laser illumination (II irradiation). (B) PA stability under prolonged pulsed laser illumination at 710 nm.

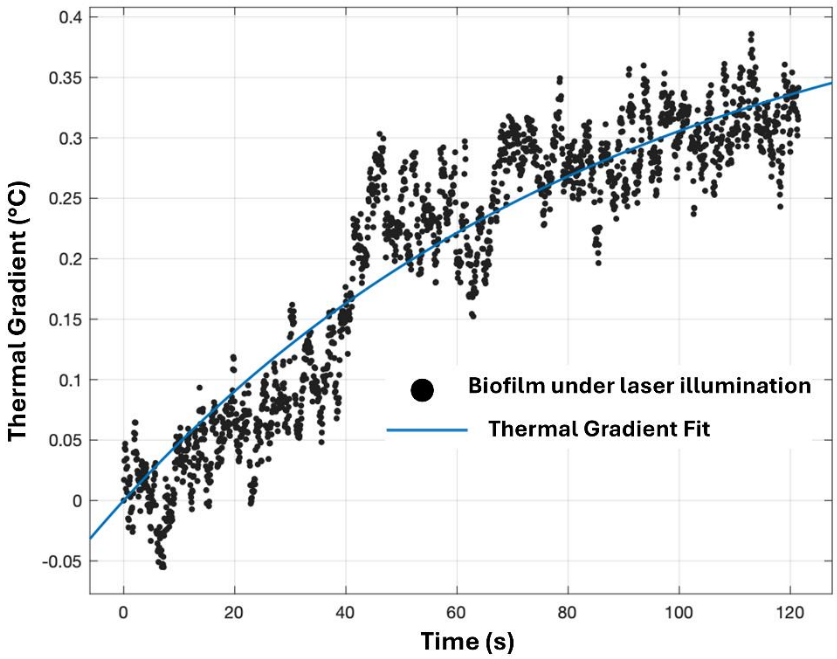

Figure S5. Photothermal activity of untreated *S. aureus* biofilm.

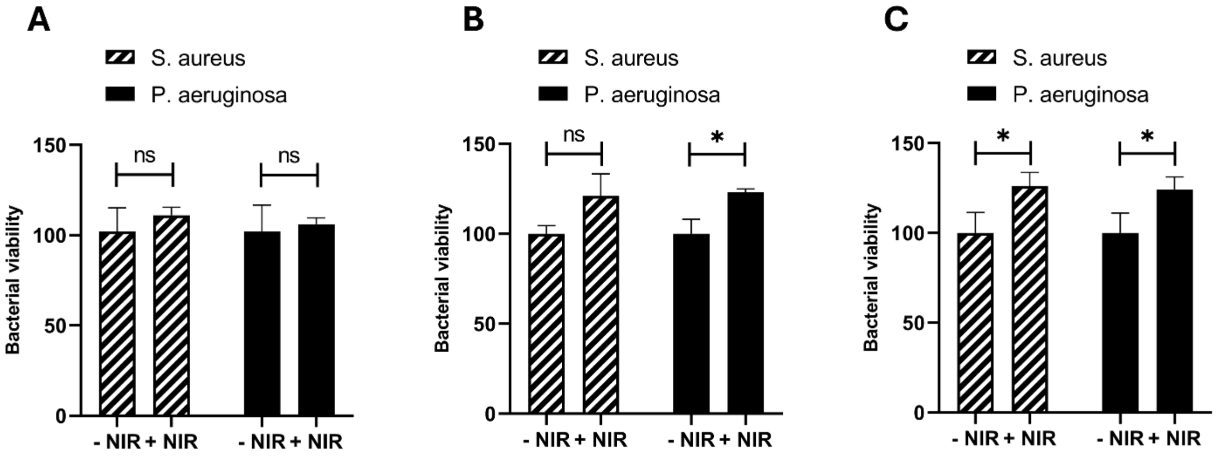

Figure S6. Viability of *S. aureus* and *P. aeruginosa* biofilms. (A) Viability of *S. aureus* and *P. aeruginosa* biofilms treated with CoPc-Lig NPs after 24h. (B-C) Viability of *S. aureus* and *P. aeruginosa* biofilms treated with NIR irradiation in the absence of NPs (B) immediately after treatment and (C) after 24h.

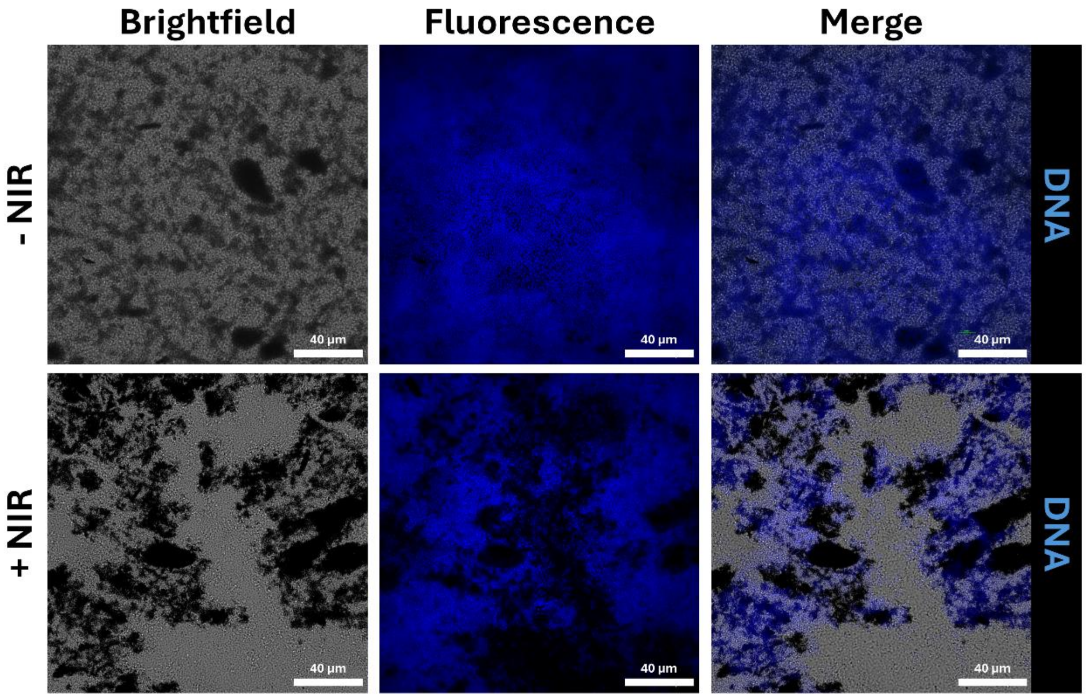

Figure S7. Enlarged confocal images showing the morphology of the S. aureus biofilm before (-NIR) and after (+ NIR) treatment with NIR irradiation. Brightfield images show CoPc-Lig NPs, fluorescence images show the bacterial DNA, and the merged images show the colocalization of NPs and bacterial cells.
